# Paternal heroin self-administration in rats increases drug-seeking behavior in male offspring via miR-19b downregulation in the nucleus accumbens

**DOI:** 10.1038/s41386-025-02081-8

**Published:** 2025-03-08

**Authors:** Wenjing Gao, Tingting Wang, Jian Cui, Nan Huang, Guangyuan Fan, Tao Pan, Changyou Jiang, Feifei Wang, Xing Liu, Lan Ma, Qiumin Le

**Affiliations:** 1 School of Basic Medical Sciences, State Key Laboratory of Medical Neurobiology, MOE Frontiers Center for Brain Science, Institutes of Brain Science, Department of Neurology, Huashan Hospital, Fudan University, Shanghai, 200032 China; 2 Research Unit of Addiction Memory, Chinese Academy of Medical Sciences (2021RU009), Shanghai, 200032 China

**Keywords:** Epigenetics and behaviour, Addiction

## Abstract

Accumulating evidence indicates that drug addiction may lead to adaptive behavioral changes in offspring, potentially due to epigenetic modifications in parental germline. However, the underlying mechanisms remain inadequately understood. In this study, we show that paternal heroin self-administration (SA) increased heroin-seeking behavior in the F1 generation, when compared with offspring sired by yoke-infused control males, indicating cross-generational impact of paternal voluntary heroin seeking behavior. Notably, the increase of heroin seeking behavior in offspring was replicated by zygotic microinjection of sperm RNAs derived from sperm of heroin-SA-experienced rats. Analysis of non-coding RNAs in spermatozoa revealed coordinated changes in miRNA content between the nucleus accumbens and spermatozoa. We validated that restoration of miR-19b downregulation in sperm RNA from self-administration-experienced rats, in parallel with its overexpression in the nucleus accumbens of F1 offspring sired by heroin-SA-experienced fathers, reversed the increased heroin SA observed in these F1 offspring. Taken together, our findings suggest in rats that paternal heroin self-administration induces epigenetic changes in both brain and sperm miRNA, with miR-19b downregulation playing a critical role in mediating the epigenetic inheritance of increased heroin self-administration behavior in the F1 generation.

## Introduction

Epidemiological studies have indicated that addiction has a heritable component, with estimates of heritability ranging from 39% to 72% [1, 2], and highlights a potential risk among offspring with a paternal and/or maternal history of drug dependence [3, 4]. Paternal opioid use in human has been linked to increased alcohol, tobacco, and marijuana use in children during early adolescence [5], as well as a higher risk of overweight in sons [6]. Studies on rodents have shown that offspring exhibit various phenotypes, including memory deficits [7], altered drug sensitivity [8, 9], and changes in sociability [10]. In our previous work using rat cocaine self-administration model, we observed contrasting behavioral outcomes in the offspring between passive drug infusion and the “addiction-like” state induced by cocaine self-administration. The latter led to a predisposition towards an addiction-vulnerable state in male offspring [11], suggesting that drug-seeking experience significantly contribute to the transmission of addiction vulnerability across generations. Yet, it remains unclear whether the conclusion could be generalized to opioids.

The impact of paternal emotional disturbances such as addiction on reproductive epigenetic modifications is a topic of interest. Previous findings demonstrate that addictive drugs results in altered acetylated histone H3 [12], DNA methylation [11, 13, 14] in the sperm. In addition, a causal relationship between changes in non-coding RNA in sperm and the epigenetic inheritance of acquired traits has been established through zygotic microinjection [15–19]. The impact of drug use on sperm RNA and its potential role in transmitting phenotypic traits across generations, has not been tested.

In this study, we used paternal heroin SA model in rats to explore the potential transgenerational effects, and examined the key sperm RNA molecules that contribute to the transmission of increased heroin-seeking behavior in the male F1 generation, suggesting a mechanism for transmitting CNS alterations to influence offspring behavior.

## Materials and methods

Experiment details, consecutively listed and labeled in Experiments 1–18, can be found in Supplementary File 1.

### Animals, housing, and breeding

Sprague-Dawley rats were housed at 23 °C, 45% humidity, in a 12-h reverse light/dark cycle (lights on at 19:00) with ad libitum food and water access. Naïve rats were purchased from the SLACCAS company, and acclimated for at least 1 week. Offspring were bred in a clean laboratory environment. Twenty-four hours after the last session, F0 rats were housed with two naïve female rats to generate F1. F2 generations were produced by crossing naïve male F1 rats with two naïve female rats. Two-to-three rats of each sex were randomly selected from each litter for tests unless otherwise specified. Procedures followed the NIH Guide for the Care and Use of Laboratory Animals and were approved by the Animal Care and Use Committee of Shanghai Medical College, Fudan University.

### Surgery

For self-administration, under isoflurane anesthesia (2.5%), rats were implanted with a catheter into the right jugular vein and connected to a back-mounted pedestal. Rats recovered for 7 days before behavioral experiments, with daily catheter flushing using 0.1 mL saline containing heparin (30 IU/mL) and gentamycin (0.5 mg/mL).

For AAV delivery, AAV_2/9_-hSyn-Cre-WPRE-pA (Taitool, China) was mixed with AAV_2/9_-flex-miR19b-EGFP or AAV_2/9_-flex-Scr-EGFP (prepared in lab) to achieve equal particle numbers. For in vivo transfection, synthetic Ago-miR-19b or scramble (Genepharma, China) was mixed 1:4 with DOTAP (Roche, Germany) and adjusted to a final concentration of 400 ng·μl^−1^. Delivery was performed at 0.24 μL·min^−1^ over 5 min using glass pipettes with Nanoject III (Drummond Scientific Co.) in NAc (AP + 2 mm; ML ± 1.2 mm; DV −7 mm).

Intracerebroventricular injection of GW4869 (50 nM in 10% DMSO, MCE, China) or vehicle was performed for four consecutive days (AP −0.8 mm; ML 1.5 mm; DV −4 mm). Tissue was harvested on the 5th day.

### Operant training and tests

Operant chambers (Med Associates, USA) were equipped with two levers, a house light, a blue cue light, and a white cue light. Pressing the active lever led to drug infusion or food/sucrose pellet delivery, accompanied by cue light and tone. Inactive lever presses had no consequences.

In sucrose self-administration tests, rats were provided with unrestricted access to food, and were trained to obtain sucrose pellets (45 mg, Bio-Serv, USA) in 1-h daily sessions. Training consisted of 5 sessions each under FR1, FR5, and FR10 schedules. Rats were then tested for one session under the PR schedule.

For food training, rats were food-restricted to 85% of their ad libitum body weight and trained to press the active lever for food pellets (45 mg, Bio-Serv) to facilitate the establishment of lever-reward associations in rats. Rats that obtained 100 pellets were then prepared for surgery.

After surgery, rats were randomly assigned to self-administer drugs or receive yoked infusions. During self-administration, two procedures were used, fixed-ratio (FR) schedule and progressive ratio (PR) tests. Rats were subjected to lever press for a given number of times (FR1, FR5, etc.) with a blue cue light signaling drug availability. For PR schedules the lever pressing response requirements increase according to the equation: i^th^ injection=Int(5e^0.25i^ - 5). Sessions ended if rats took more than 1 h to meet the requirement. The infusions under PR schedule, the last lever press required for an injection, was recorded. For yoked infusions, while one rat could press for heroin or saline, a paired rat received passive infusions at the same dose, time, and rate. Lever presses by yoked rats had no effect.

For F0, 5-day FR1, 25-day FR5, 1-day PR was used. For F1 generation, 3-day FR1, 6-day FR5, 1-day PR was used. Drug was delivered at 45 μg·kg·inf^-1^ over 4 s. For FR, sessions consisted of three 40-min drug-available periods dispersed by two 15-min no-drug periods within a 2.5-h window, distinguished by house light. In dose-response curve tests, rats were trained to press levers for decreasing doses of heroin (67.5, 45, 27, 20, 6.75, 4.5, 2, 0 μg/kg, i.v.) under FR5. Each dose was maintained until responses stabilized over 2 days (<10% variation). Mean lever presses were calculated from the final 2 days of each dose.

### Behavioral tests on anxiety, short-term memory, and sociability

Three days prior to the tests, the rats were acclimated to the test room for 1 h per day. The order was as follows: open field test (OFT), elevated plus maze test (EPM), light/dark box test (LDB), sociality test, and Y-maze test. In OFT chambers with a light intensity of 60 lux, distance traveled, center time, and entries of each rat were recorded for 30 min. For EPM test, rats were placed in an apparatus elevated 50 cm high, had two closed and two open arms for 6 min, with time in open arms recorded and analyzed using Clever System software. In LDB tests, rats were placed in an apparatus comprising a black and a transparent chamber and allowed to freely explore both sides for 15 min.

Sociability and social recognition tests were performed in an apparatus comprised two end chambers and a central chamber separated by arched doors. Sociability test was performed in the presence of an empty cage and a cage containing Stranger A. To assess social recognition, Stranger B was then introduced, and the test rat explored for another 10 min. The time spent exploring the cages and interacting with the strangers were recorded.

Y-maze was constructed of beige Plexiglas with three arms at 120° angles. During training, rats explored the maze for 10 min with one arm blocked. One hour later, rats were re-introduced into the maze with all arms were accessible for 5 min. Novelty preference=$\frac{{{{\rm{time}}}}\; {{{\rm{spent}}}}\; {{{\rm{in}}}}\; {{{\rm{novel}}}}\; {{{\rm{arm}}}}}{{{{\rm{time}}}}\; {{{\rm{spent}}}}\; {{{\rm{in}}}}\; {{{\rm{all}}}}\; {{{\rm{arms}}}}}\times 100 \%$.

### Zygote collection and sperm RNA microinjection

Five-week-old virgin female rats were superovulated with PMSG and HCG, and housed with a naïve male SD rat. Zygotes were collected from plugged females by flushing from the fimbriae tubae, and then used for RNA injection into the cytoplasm. Pseudopregnant female rats, confirmed by copulatory plugs from mating with vasectomized males, were used as recipients. Sperm RNA was adjusted to a concentration of 1 ng/μL, and injected into the cytoplasm if zygotes. Approximately 20 zygotes were transferred into one side of the oviduct.

### Tissue collection

Rats were euthanized with isoflurane overdose and perfused with cold PBS. Brains were removed, sliced into 1 mm sections for NAc dissection. Cauda epididymal spermatozoa were released, purified by swim-up, lysed in a somatic cell lysis buffer (0.1% SDS, 0.5% Triton X-100). The pellet containing spermatozoa was then Percoll purified, visually inspected, and stored in Trizol reagent.

### RNA extraction and qPCR

RNA was extracted using the Direct-zol® RNA Microprep Kit. Sperm samples were checked with Agilent RNA Pico chip to be with <0.5% ribosomal contamination. RNA from somatic regions should be with RIN ≥ 8. Canonical reverse transcription was performed using the HiScript II 1st Strand cDNA Synthesis Kit (Vazyme). Synthetic miRNA mimics (GenePharma Co., Ltd., China) were diluted to 1 pM and reverse transcribed into cDNA to generate a standard curve for absolute quantification of miRNAs. For miRNAs, the S-Poly(T) Plus method [20] was used. Real-time quantitative PCR was done at 95 °C 5 min, followed by 40 cycles of 95 °C for 10 s and 60 °C for 30 s (CFX Opus 384, Bio-Rad). Reactions were run in triplicate.

### Molecular cloning

Cre-dependent miRNA overexpression or knockdown was achieved by cloning synthetic miR-30-based scaffolds, or decoy TuD RNA using EcoRI/XhoI restriction sites into the pAKD-CMV-bGlobin-Flex-EGFP-MIR30shRNA vector. For luciferase assay, oligonucleotides encoding miR-19b target sites or scramble sequences were inserted into XhoI/NotI-digested psiCHECK-2 (Promega).

### Luciferase assay

For the luciferase assay, the pSICHECK2-miR-19b or a scramble control plasmid co-transfected with synthetic miR-19b into HEK-293T cells. Twenty-four hours post-transfection, the Dual-Luciferase^®^ Reporter Assay System (Promega) was utilized.

### Library preparation, sequencing, and bioinformatic analysis

Total RNA library was prepared using the Ribo-off rRNA Depletion Kit and VAHTS Universal V8 RNA-seq Library Prep Kit for Illumina (Vazyme) with 100 ng of total RNA. These libraries were sequenced on the NovaSeq 6000 for ~50 million reads. Raw reads were subjected to cleaning using Trimmomatic (V0.32) [21], and aligned to rat genome (Ensembl Rnor_6.0) using Hisat2 (V2.0.2) [22]. Gene annotation and read counting were performed using featureCounts [23].

Small RNA library preparation was done using the VAHTS^TM^ Small RNA Library Prep Kit for Illumina (Vazyme LLC, China), with 200 ng of total RNA. Sequencing was carried out on either the Illumina MiSeq or NovaSeq 6000 for ~10 million reads per sample. Reads were cleaned with NGmerge [24], and analyzed using Sports1.0 [25].

A network propagation-based model [26] was used to predict regulating miRNAs of potential addiction-related genes [27]. Network propagation enrichment score (NPES) for each microRNA were extracted for ranking.

Differential expression analyses were done using DESeq2 [28]. Significance was set at an adjusted p-value of 0.05. Heatmaps were plotted using “pheatmap” (V1.0.12). Gene ontology annotation was performed using Enrichr [29] and clusterProfiler [30]. The concordance of miRNA expression signature was evaluated using RRHO2 (V1.0) [31].

### Statistical analysis

Statistics were performed with using R (V4.2.2), plotting were done with R or GraphPad Prism (V9.0.0). Linear mixed-effects model was used, and *post hoc t*-tests were done with Satterthwaite’s method using packages “lme4” (V1.1-35.5), “lmertTest” (V3.1-3) and “mice” (V3.17.0). All statistical details can be found in Supplementary Table 1 (for raw data and statistical results) and Supplementary File 2 (to reproduce the statistical analysis and generate report for each figure). *P* < 0.05 was considered statistically significant. The data are presented as mean ± standard error of the mean (SEM).

## Results

### Paternal heroin self-administration increases heroin seeking in male F1 offspring compared with saline- and yoked-sired controls

**Fig. 1:**
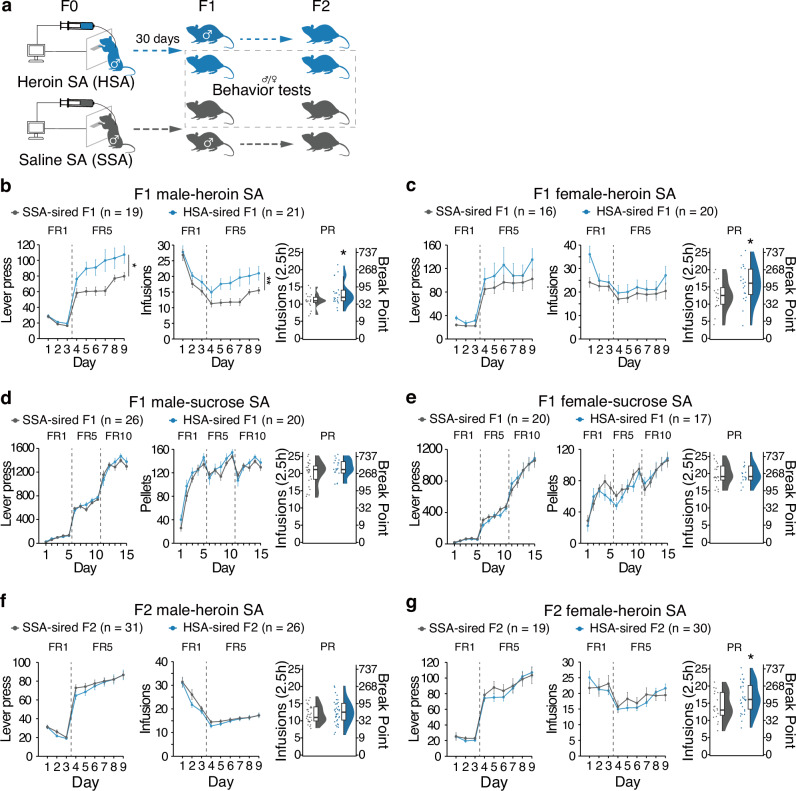
Effects of paternal heroin self-administration on heroin and sucrose self-administration behavior in offspring. **a** Mating and behavioral assessment schedule. Rats trained to self-administer heroin (45 μg/kg/inf, HSA) or saline (SSA) were mated with naïve female rats, and offspring generated were subjected to behavioral tests. F2 was obtained by mating random F1 male rats with naïve female rats. **b** Lever pressed in FR sessions, infusions in FR sessions, and infusions in PR session in heroin SA tests of F1 male offspring. **c** Lever pressed in FR sessions, infusions in FR sessions, and infusions in PR session in heroin SA tests of F1 female offspring. **d** Lever pressed in FR sessions, pellets gained in FR sessions, and infusions in PR session in sucrose SA tests of male F1 offspring. **e** Lever pressed in FR sessions, pellets gained in FR sessions, and infusions in PR session in sucrose SA tests of female F1 offspring. **f** Lever pressed in FR sessions, infusions in FR sessions, and infusions under PR schedule in PR session in heroin SA tests of F2 male offspring. **g** Lever pressed in FR sessions, infusions in FR sessions, and infusions in PR session in heroin SA tests of F2 female offspring. For FR tests, results are presented as mean ± s.e.m in the line plot. For PR tests, polygon represents density estimates of data, the box represents the 25th and 75th percentiles, the whiskers show the minimum and maximum of the data, and the line inside the box denotes the median. *P < 0.05, ***P < 0.001.

Naïve male SD rats were allowed to self-administer heroin (HSA group) or saline (SSA group) on a FR schedule for 30 days. On day 31, they were subjected to a PR test (Fig. S1a–c), and mated with naïve females to produce offspring (Fig. 1a). Male F1 offspring sired by heroin-SA-experienced fathers showed higher lever presses for heroin (*F*_group_(1, 39.9) = 5.199, *P* = 0.028), higher infusions in FR (*F*_*group*_(1, 38) = 9.18, *P* = 0.00439) and increase of infusions under PR schedule (*F*_group_(1, 38) = 4.87, *P* = 0.0335), compared with saline-sired F1 (Fig. 1b). In female offspring, no significant difference was found under FR (lever, *F*_group_(1, 34.8) = 0.718, *P* = 0.403; infusions, *F*_group_(1, 34) = 1.273, *P* = 0.267), but there was an increase of infusions under PR schedule (*F*_group_(1, 34) = 4.19, *P* = 0.0485), compared with saline-sired progeny (Fig. 1c).

As significant individual differences in abuse potential have been reported in both human [32] and rodent models [33], we examined whether the pattern of paternal drug-seeking behavior would affect their offspring (Fig. S1d). For a random cohort of 16 SD rats and their offspring, the lever-pressing performance was compared. On day 9 of SA schedule, the lever presses of heroin-experienced F1 generation were significantly higher than that of F0, which restated that paternal heroin-seeking increased heroin seeking in F1 generation (Fig. S1e). Using principal component analysis (PCA), we roughly divided F0 into three clusters: Hi-SA, Med-SA, and Lo-SA (Fig. S1f). Although the three groups showed significant differences in lever pressing during FR (Fig. S1f), no-drug periods (Fig. S1i), and PR tests (Fig. S1l), their offspring performed similarly (Fig. S1g, j, m). No significant correlation was observed in the standardized scores for FR, no-drug period lever pressed, or PR between F0 and F1 (Fig. S1h, k, n). Furthermore, we observed comparable performance in sucrose SA (Fig. 1e–f. Male: FR, *F*_group_(1, 21) = 0.171; pellets, *F*_group_(1, 21) = 0.801, *P* = 0.381; infusions under PR, *F*_group_(1, 44) = 2.34, *P* = 0.133. Female: FR, *F*_group_(1, 17.2) = 0.016, *P* = 0.9; pellets, *F*_group_(1, 17.212) = 0.219, *P* = 0.646; infusions under PR, *F*_group_(1, 16.9) = 0.005, *P* = 0.946), distance traveled (male, *t*(14.5) = -0.174, *P* = 0.864; female, *t*(16.5) = 0.937, *P* = 0.326), time spent in the center area (male, *t*(14.5) = −0.174, *P* = 0.864; female, *t*(16.5) = 0.937, *P* = 0.362) and entries to the center (male, *t*(14.5) = −0.174, *P* = 0.864; female, *t*(16.5) = 0.937, *P* = 0.362) of OFT test, time in the light box of LDB test (male, *t*(36.0) = −0.757, *P* = 0.454; female, *t*(15.7) = −0.483, *P* = 0.636), time in the open arm of EPM test (male, *t*(13.4) = −1.139, *P* = 0.275; female, *t*(15.7) = −0.483, *P* = 0.636), ratio in novel arm of Y-maze (male, *t*(36.0) = 1.059, *P* = 0.297; female, *t*(15.8) = −0.587, *P* = 0.565), sociability tests (male, *F*_group×test_(1, 72) = 2.73, *P* = 0.103; female, *F*_group×test_(1, 70) = 0.128, *P* = 0.722) or social recognition tests (male, *F*_group×test_(1, 72) = 0.746, *P* = 0.39; female, *F*_group×test_(1, 70) = 1.126, *P* = 0.292), between the two groups in both sexes (Fig. S2).

Naïve male F1 rats were co-housed with naïve females to produce F2 offspring (Fig. 1a). Both male and female F2 offspring from these two groups exhibited similar lever pressing for heroin under the FR schedule (Fig. 1f-g. FR, male, *F*_group_(1, 69.6) = 0.517, *P* = 0.475; female, *F*_group_(1, 21.8) = 0.269, *P* = 0.609. Infusions, male, *F*_group_(1, 67) = 1.14, *P* = 0.29; female, *F*_group_(1, 28.8) = 0.036, *P* = 0.852), but female F2 offspring sired by heroin-SA experienced grandfathers had a higher infusions under PR schedule (male, *F*_group_(1, 24.2) = 0.918, *P* = 0.347; female *F*_group_(1, 47) = 4.14, *P* = 0.0476). This suggests paternal heroin reinforcement may lead to transgenerational epigenetic inheritance, and significant sex difference in behavior.

**Fig. 2:**
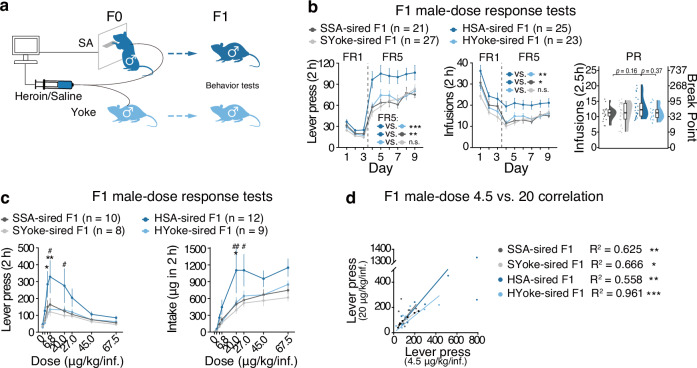
The inheritance of increased heroin self-administration behavior is contingent on paternally motivated voluntary administration of heroin. **a** Naive male rats were randomly paired. In each pair, one rat was allowed to self-administer heroin or saline by pressing the lever (SA rat), while the other passively received the same dose of infusion at the same time when the SA rat pressed the lever (yoked rat). Pairs of Her and Sal groups were used to generate F1 offspring. **b** Lever pressed in FR sessions, infusions in FR sessions, and infusions in PR session in heroin SA tests of male F1 offspring. **c** Lever pressed and dosage of drug obtained in heroin dose-response teste of F1 offspring. *, HSA-sired F1 vs. SSA-sired F1. #, ##, HYoke-sired F1 vs. HSA-sired F1. **d** Correlation of lever press at heroin dose of 4.5 μg/kg/inf and 20 μg/kg/inf of F1 offspring. For FR tests, results are presented as mean ± s.e.m in the line plot. For PR tests, polygon represents density estimates of data, the box represents the 25th and 75th percentiles, the whiskers show the minimum and maximum of the data, and the line inside the box denotes the median. * or # *P* < 0.05, ** or ## *P* < 0.01, ***P < 0.001.

Additionally, we conducted a yoked heroin infusion experiment, pairing each SA rat with a yoked control receiving the same dose of drug at the same time (Fig. 2a). Male F1 offspring sired by heroin-SA-experienced fathers showed significantly higher FR5 lever presses than saline-sired F1 rats (lever, *F*_drug×deliveryMethod_(1, 29.3) = 2.37, *P* = 0.135; FR5, ***P = 0.00018; infusions, *F*_drug×deliveryMethod_(1, 29.7) = 3.16, *P* = 0.0857; FR5 **P = 0.0081), while under yoked infusions, paternal heroin infusions did not induce significant changes in F1 offspring (lever, FR5, *P* = 0.91019; infusions, *P* = 0.7521, Fig. 2b). We failed to grasp a significant difference in infusions under PR schedule between the groups (Fig. 2b, *F*_drug×deliveryMethod_(1, 32.3) = 3.45, *P* = 0.0724). Male F1 offspring from heroin SA-experienced fathers had higher lever presses than other F1 offspring at various doses (Fig. 2c, lever, *F*_drug×deliveryMethod_(1, 35.0) = 1.63, *P* = 0.210; 4.5 μg*kg/inf, HSA-sired F1 *vs*. HYoke-sired F1, *P = 0.0192; 6.8 μg*kg/inf, HSA-sired F1 *vs*. SSA-sired F1, *P = 0.0253, HSA-sired F1 *vs*. HYoke-sired F1, **P = 0.0084; 20 μg*kg/inf, HSA-sired F1 *vs*. SSA-sired F1, *P = 0.0494). Positive correlations of lever presses at 4.5 μg*kg/inf and 20 μg*kg/inf doses were observed in all groups (Fig. 2d), with similar coefficients (HSA-sired F1 *vs*. SSA-sired F1, *Z* = −0.212, *P* = 0.832; HSA-sired F1 *vs*. HYoke-sired F1, *Z* = 1.512, *P* = 0.131). This suggests an upward shift in the dose-response curve for male F1 offspring sired by heroin self-administration-experienced fathers, likely due to increased motivation rather than enhanced sensitivity to heroin. Overall, these findings indicate that paternal drug-seeking behavior for heroin reinforcement, rather than heroin exposure itself, is crucial for increased heroin self-administration in offspring.

### Alterations of sperm RNA are causal to the increased heroin self-administration phenotype in offspring

**Fig. 3:**
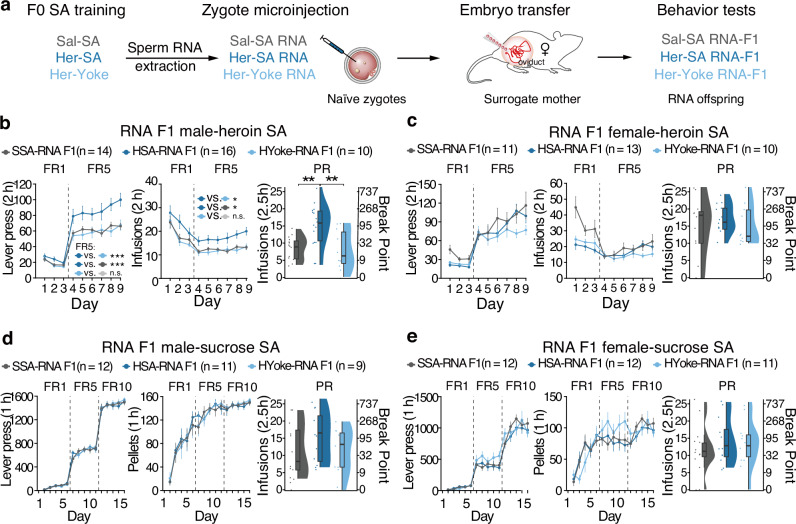
Alterations in sperm RNA are causally linked to increased heroin self-administration in male F1 offspring. **a** Experimental scheme. Total RNAs purified from sperm of HSA, HYoke, or SSA F0 generation were microinjected into naïve fertilized oocytes and then transferred into the oviduct of surrogate rats. **b** Lever pressed in FR sessions, infusions in FR sessions, and infusions in PR session in heroin SA tests of RNA male offspring. **c** Lever pressed in FR sessions, infusions in FR sessions, and infusion in PR session in heroin SA tests of RNA female offspring. **d** Lever pressed in FR sessions, pellets gained in FR sessions, and infusions in PR session in sucrose SA tests of RNA male offspring. **e** Lever pressed in FR sessions, pellets gained in FR sessions, and infusions in PR session in sucrose SA tests of RNA female offspring. For FR tests, results are presented as mean ± s.e.m in the line plot. For PR tests, polygon represents density estimates of data, the box represents the 25th and 75th percentiles, the whiskers show the minimum and maximum of the data, and the line inside the box denotes the median. **P < 0.01.

Increasing evidence suggests that alterations in sperm epigenetic signatures, including non-coding RNAs, correlate with phenotypic changes in subsequent generations. We isolated total RNA from sperm of HSA, HYoke, and SSA groups. This RNA was then microinjected into naïve fertilized oocytes (Fig. 3a). The resulting HSA-RNA F1 male exhibited significantly higher lever presses (*F*_*group*_(2, 38.0) = 4.6, *P* = 0.0163; FR5, HSA-RNA F1 *vs*. SSA-RNA F1, ***P = 4.00e-4; HSA-RNA F1 *vs*. HYoke-RNA F1, ***P = 0.000406), and received more infusions (*F*_*group*_(2, 37.0) = 5.97, *P* = 0.00566; HSA-RNA F1 *vs*. SSA-RNA F1, *P = 0.0178; HSA-RNA F1 *vs*. HYoke-RNA F1, *P = 0.0146) for heroin in FR, and higher infusions under PR schedule compared with the F1 offspring from saline-SA-experienced or yoke heroin-infused fathers (Fig. 3b, *F*(2, 37) = 7.936, *P* = 0.00136; HYoke-RNA F1 vs.HSA-RNA F1,**P = 0.00556; SSA-RNA F1 vs.HSA-RNA F1,**P = 0.00532), but in female, no significant difference was observed (Fig. 3c, lever, *F*_*group*_(2, 32.1) = 1.40, *P* = 0.262; infusions, *F*_*group*_(2, 31) = 3.31, *P* = 0.0499; infusions under PR, *F*(2, 31) = 0.5, *P* = 0.612). Furthermore, there were no notable differences in sucrose self-administration behavior among the groups across both sexes (Fig. 3d-e. Male, lever, *F*_*group*_(2, 29.0) = 0.004, *P* = 0.996; pellets, *F*_*group*_(2, 29) = 0.003, *P* = 0.997; infusions under PR, *F*_*group*_ (2, 29) = 0.995, *P* = 0.382. Female, lever, *F*_*group*_(2, 32) = 0.493, *P* = 0.616; pellets, *F*_*group*_(2, 32) = 0.277, *P* = 0.76; infusions under PR, *F*_*group*_ (2, 32) = 0.284, *P* = 0.755). These data support a causal role for alterations in sperm RNA in the development of increased heroin self-administration behavior in male offspring.

### Alterations of miRNA expression profiles in sperm and brain after heroin SA are correlated

**Fig. 4:**
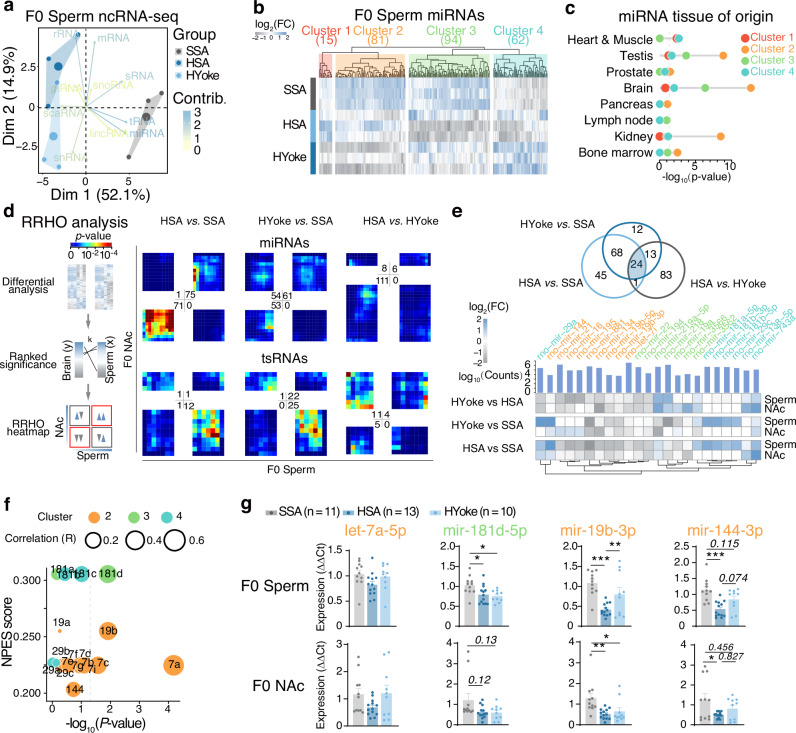
Correlation of miRNA expression patterns in sperm and the nucleus accumbens of the F0 generation. **a** Principal component analysis of F0 sperm non-coding RNA profiles. Arrows indicate the contribution of each non-coding RNA type to each dimension. **b** Heatmap and hierarchical clustering of differentially expressed miRNAs between SSA, HSA and HYoke of F0 sperm. **c** Cellular component enrichment of the four clusters of differentially expressed sperm miRNAs. **d** Rank Rank Hypergeometric Overlap (RRHO) analysis of differentially expressed miRNAs and tsRNAs in the NAc vs. sperm of the F0 generation. The upper-right and lower-left region of each plot represents co- up/down regulation, while the upper-left and lower-right regions shows inconsistent changes. Color indicates significance. **e** Overlaps of HYoke vs. SSA, HSA vs. SSA, HSA vs. HYoke. Venn diagram (top) and heatmap (bottom) of candidate miRNAs. The heatmap shows the log_10_(Counts) of each miRNA in F0 sperm (bars) and the relative fold change of each miRNA in both the sperm and the nucleus accumbens of F0 generation was shown. Colors of miRNA names correspond to the clustering of the miRNA in (**b**). **f** Correlation plot. Significance of correlation between sperm and NAc of F0 HSA group (−log_10_(*P*-value)) *vs*. the enrichment score of addiction-related miRNA targets (NPES score). Size represents Pearson correlation coefficient of miRNA expression level between sperm and NAc of F0 HSA group. Colors correspond to the clustering of the miRNA in (**b**). **g** Relative expression of candidate miRNAs in sperm and NAc of the F0 generation. Results are shown as mean ± s.e.m. *P < 0.05, **P < 0.01, ***P < 0.001.

We performed small RNA sequencing on sperm of F0 generation. PCA revealed key contributions to the differences of miRNAs, tRNAs, and rRNAs (Fig. 4a). Furthermore, analysis of RNA content revealed a notable depletion of sperm miRNAs in heroin self-administration experienced rats compared with the saline control group (Fig. S3a–c, log_2_(fold change) = −3.54, *P* = 2.62e-9). Consequently, we focused our further investigations on the differences in miRNA expression.

Hierarchical clustering of differentially expressed miRNAs revealed four distinct clusters of expression patterns (Fig. 4b). Clusters 1 and 3 enriched miRNAs significantly upregulated/downregulated in the sperm of heroin-SA experienced rats, respectively. Cluster 2 contained miRNAs downregulated in response to heroin exposure. In contrast, Cluster 4 was enriched with miRNAs that were inversely expressed in HSA and HYoke groups compared to SSA. Using miRNA set enrichment analysis tool (TAM 2.0) [34], we discovered that altered sperm miRNAs were enriched particularly in the brain, in Clusters 2, 3, and 4 (Fig. 4c). Similarly, miEAA indicated Cluster 3 originated from the cerebrum-thalamus and pituitary (Fig. S3d), and unveiled distinct overrepresented pathways (Fig. S3e). Then we included NAc and mPFC for small RNA-seq, and surprisingly found, using RRHO analysis, a positive correlation between changes of sperm and NAc miRNA profiles (Fig. 4d, upper), but not in tsRNAs (Fig. 4d, lower). Furthermore, the correlation was not found in the mPFC vs. sperm (Fig. S4g). These observations implicate a potential association between heroin-induced epigenetic remodeling of miRNA profiles in NAc brain region and changes in sperm.

By comparing differentially expressed sperm miRNAs among the three groups, we identified 28 core miRNAs with consistent significant alterations in sperm and the NAc (Fig. 4e) from the sequencing data. We also performed transcriptome sequencing in the NAc of F1 (Fig. S4a–b), and confirmed that these core miRNAs indeed significantly modulate gene expression differences in NAc (Fig. S4c). We ranked these core miRNAs for their potential to target addiction-regulating genes using a network propagation strategy [35]. The top-ranked miRNAs-miR-144, let-7 family, miR-19b (Cluster 2), miR-19a (Cluster 3), and miR-181a/b, miR-29a/c (Cluster 4) were selected for further validation in a separate batch of samples of F0 and their F1 offspring. Although we observed some considerable inconsistencies in the HYoke group, compared with sequencing results, the high correlation coefficients between sperm and NAc persisted (Fig. 4f, Fig. S5). Among these, miR-19b exhibited the strongest significance (Fig. 4g) (*F*(2, 31) = 6.041, *P* = 0.006, SSA *vs*. HSA, **P = 0.007; SSA vs HYoke, *P = 0.041) and was altered in the NAc (Fig. S4d, *F*(2, 9) = 10.01, *P* = 0.00515, male, HSA-RNA F1 *vs*. SSA-RNA F1, **P = 0.00215, HYoke-sired-F1 *vs*. SSA-sired-F1, **P = 0.00381; female, HSA-RNA F1 *vs*. SSA-RNA F1, *P = 0.0394) and sperm of F1 offspring sired by heroin-SA experienced fathers (Fig. S4e, F(2, 24) = 5.073, *P* = 0.015; SSA-sired-F1 *vs*. HSA-sired-F1, *P = 0.012). The results of the PCR study indicated the necessity of further investigation into the role of miR-19b in the mediation of trait transmission and the regulation of heroin SA behavior.

### MiR-19b is critically involved in the epigenetic transmission and the increased heroin SA in Heroin F1 offspring

**Fig. 5:**
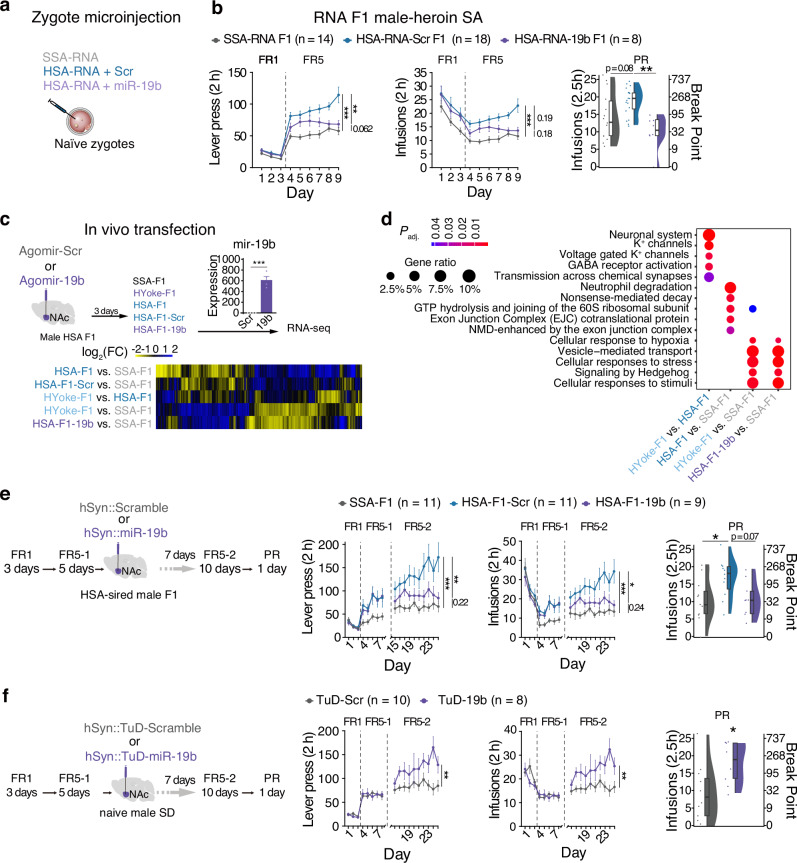
MiR-19b is critical for the epigenetic inheritance and the increased heroin self-administration phenotype in F1 offspring. Schematics (**a**) and heroin self-administration tests (**b**) on RNA offspring obtained by microinjection of RNA from SSA-RNA (SSA-RNA-F1), HSA-RNA + Scr-RNA (HSA-RNA-Scr-F1), miR-19b-normalized HSA-RNA (HSA-RNA-19b-F1) into naïve fertilized oocytes were tested. **c** In vivo transfection of candidate miRNAs and RNA-seq of the transfected NAc. Upper, Agomir-Scr, or Agomir-19b were in vivo transfected in the NAc of HSA-F1, validated for target miRNA overexpression, and then subjected to RNA-seq. Lower, heat map of differentially expressed genes between groups. **d** Differential overrepresentation of pathways in agomir-transfected F1. **e** Performance of heroin SA behavior before and after miR-19b over-expression in the NAc of male HSA-sired F1 generation. **f** Performance of heroin SA behavior before and after miR-19b knockdown with TuD-miR-19b in the NAc. For FR tests, results are presented as mean ± s.e.m in the line plot. For PR tests, polygon represents density estimates of data, the box represents the 25th and 75th percentiles, the whiskers show the minimum and maximum of the data, and the line inside the box denotes the median. *P < 0.05, **P < 0.01, ***P < 0.001.

To establish a causal relationship between miR-19b downregulation in sperm of heroin-SA experienced rats and increased heroin self-administration in their offspring, we supplemented sperm RNA of heroin-SA experienced rats with synthetic miR-19b to match levels of saline-SA rats (Fig. S6) and microinjected these into naive fertilized oocytes (Fig. 5a). Normalizing miR-19b levels reduced heroin self-administration behavior of the male HSA-RNA F1 group (Fig. 5b, lever, *F*_group_(2, 38.3) = 8.64, *P* = 0.000798; FR5, HSA-RNA+19b F1 *vs*. HSA-RNA F1, **P = 0.00327; infusions, *F*_group_(2, 37) = 9.561, *P* = 0.000449; HSA-RNA+19b F1 *vs*. HSA-RNA F1, *P* = 0.194; infusions under PR schedule, *F*_Group_(2, 37) = 7.867, *P* = 0.00142; HSA-RNA+19b F1 vs. HSA-RNA F1,**P = 0.00135), suggesting the critical role of miR-19b in the epigenetic transmission of heroin-induced changes.

Next, we transfected miR-19b in vivo into the NAc of F1 offspring sired by heroin self-administration-experienced fathers and extracted RNA samples for expression analysis and sequencing (Fig. 5c). Both heatmap and pathway overrepresentation analyses confirmed that miR-19b overexpression shifted its transcriptome profiles toward those of F1 sired by yoke heroin-infused fathers, indicating miR-19b’s crucial role in mediating changes induced by paternal heroin-seeking behavior (Fig. 5c, d).

Then, male F1 rats sired by heroin self-administration-experienced fathers were split into two groups based on equal lever pressure after initial heroin-SA training, and received viral injection into the NAc to overexpress miR-19b or miR-Scramble (Fig. 5e, S7). We found that viral expression of miR-19b, unlike the scramble control, significantly attenuated the escalation of lever presses for heroin in the FR5 schedule (*F*_group_(2, 28.9) = 4.28, *P* = 0.0236; FR5-2, **P = 0.00867), down-regulated drug infusions (*F*_group_(2, 29.0) = 4.08, *P* = 0.0276; FR5-2, **P = 0.0178), and the infusions under PR schedule was indifferent compared with SSA-sired F1 (*F*_group_ (2, 28) = 4.46, *P* = 0.0209; HSA-F1-AAV-19b *vs*. SSA-sired F1, *P* = 1, Fig. 5e). Conversely, we constructed a miRNA sponge (TuD) to antagonize miR-19b (Fig. S8a, b). Naive rats were trained to self-administer heroin and randomly assigned to express TuD-miR-19b or TuD-Scramble in the NAc (Fig. 5f). Inhibition of miR-19b expression decreased lever presses for heroin (*F*_group_(1, 16.7) = 1.38, *P* = 0.257; FR5-2, **P = 0.00229), and exhibited a trend of decrease in number of daily drug infusions (*F*_group_(1, 16) = 3.61, *P* = 0.0759; FR5-2, *P* = 0.0759) after viral delivery, and down-regulated the infusions under PR schedule in the PR schedule, in contrast to the injection of scramble-TuD (*t*(15.9) = −2.643, *P = 0.0178). In summary, these results establish a causal link between deregulation of miR-19b and increased heroin self-administration behavior in F1 offspring, supporting the role of miR-19b in mediating increased heroin-seeking behavior and participating in cross-generational epigenetic transmission.

## Discussion

In this study, we show that paternal heroin self-administration in rats results in increased heroin-seeking behavior in F1 male offspring. This effect was replicated by zygotic microinjection of sperm RNAs from heroin self-administration-experienced rats, but not from yoked infusion pairs, highlighting the role of sperm non-coding RNAs changes induced by paternal drug-seeking behavior. Analysis of non-coding RNA changes in the NAc and sperm of the F0 generation revealed a significant correlation in miRNA expression profiles, particularly the downregulation of miR-19b in both tissues, which was linked to the observed phenotype. The heightened heroin-seeking behavior in the male F1 generation could be reversed by supplementing synthetic miR-19b in F0 sperm RNA or introducing miR-19b into the NAc of F1 offspring. These findings suggest that sperm miRNAs like miR-19b mirror changes in brain miRNAs, participate in epigenetic transmission of acquired traits from F0 to F1, as well as in regulating heroin SA behavior of offspring.

### Effects of paternal voluntary heroin seeking on drug-seeking behaviors of offspring

Epidemiological studies have shown significant familial transmission of substance use disorders [36, 37]. In addition, animal studies have shown that paternal exposure to reinforcers, such as cocaine [38], morphine [39], THC [40], and even palatable food [41] can induce various developmental and physiological abnormalities in the offspring [42]. In our previous work, we found that paternal passive infusions of cocaine and the “addiction-like” state induced by cocaine seeking elicit contrasting behavioral outcomes in offspring, with the latter increasing the vulnerability of male offspring to develop cocaine-seeking behavior [11]. Here using heroin model, it is imperative to reiterate the assertion that the voluntary drug-seeking experience exerts a more substantial influence on the offspring’s motivation to seek substances of exposure in comparison to mere exposure to drugs.

Endogenous opioids play a major role in stress management, cognition, mood, and pain perception in the central nervous system, while exerting endocrine [43], immune [44], and metabolic effects [45]. Notably, opioid peptides like endorphins and enkephalins have been detected in seminal fluid and testicular cells [46], and mu-, delta-, and kappa-opioid receptors have been identified in mouse [47] and human sperm cells [48]. Furthermore, morphine administration could lead to an increased number of immotile spermatozoa, consistent with clinical findings that heroin and methadone users have decreased sperm motility [49]. This highlights the possibility that exposure to exogenous opioids may thus disrupt multiple homeostatic mechanisms, including the reproductive system. How opioids, which exert effects outside the central nervous system while contributing to addiction within the CNS, interact with and impact the reproductive system remains unclear and warrants further investigation.

In this study, we investigated the effects of paternal heroin exposure on various behaviors in the offspring, including OFT, EPM, sucrose water consumption, sociability, and Y-maze, etc., but it is surprising that no significant alterations were observed in other behaviors. In addition, our previous research [8] shows no significant differences in body weight, litter size, object-based attention tests, or cocaine self-administration between the HSA-sired and SSA-sired F1 generations in either sex. However, we observed a significant increase in the analgesic effect of heroin in male F1 offspring sired by heroin self-administration-experienced fathers during the hot plate test, demonstrating that paternal heroin exposure primarily influences heroin-related behaviors, particularly in male offspring. Notably, inter-individual variability within our dataset may contribute to the lack of statistically significant differences in most of the behaviors assessed. Furthermore, differences in the effects of heroin-seeking experiences on offspring behaviors were found between species, between models, and even between rats and mice, highlighting the importance of species-specific factors in these studies. Overall, our findings highlight the need for further research to elucidate the underlying mechanisms and to improve our understanding of how paternal behavior and exposure may influence offspring development.

A large high-throughput study has shown that a significant proportion of mammalian traits are influenced by sex [50]. While recent conclusions suggest that there are no substantial sex differences in the reinstatement of drug seeking or the incubation of craving for opioids [51], transgenerational epigenetic inheritance models show differential responses between the sexes in the offspring. Our research revealed nuanced differences in how paternal heroin-seeking behavior is imprinted in offspring of different sexes. Specifically, male F1 offspring showed a marked increase in drug-seeking behavior that did not significantly persist in the F2 generation. Conversely, female offspring showed increased motivation for drug seeking in both the F1 and F2 generations, as evidenced by their performance in the progressive ratio procedure. These differences highlight the need for a deeper understanding of the biological mechanisms governing sex dimorphism in non-Mendelian inheritance. Hormonal influences, imprinted epigenetic marks, and sex chromosomes have been implicated in these mechanisms [52].

### The role of miRNAs in paternal epigenetic transmission of acquired traits

In recent research on the mechanism of cross-generational inheritance of an acquired trait, particular attention has been directed towards sperm-borne non-coding RNAs as vehicles of epigenetic information. In our study, we found that the increased heroin self-administration behavior in male offspring was recapitulated by zygotic microinjection of sperm RNA from Heroin F0 generation, suggesting the key role of sperm RNA in the epigenetic inheritance of increased heroin self-administration phenotype. Recent investigations highlight the pivotal role played by sperm non-coding RNAs, notably miRNA [18], mitochondrial RNAs [53], transfer RNA-derived small RNAs (tsRNA) [54], and long noncoding RNA [15], in mediating the inheritance of environmentally influenced traits [55]. Of note, increasing evidences have identified miRNA in sperm as an important mediator in the inheritance of emotional perturbations, such as stress [17], depression [18], and environment enrichment [19]. Rodgers et al. showed that microinjection of nine miRNA compounds into normal zygotes induced blunted HPA axis activation on offspring and reduction of stored maternal mRNA transcripts [17]. Wang et al. found that injection of synthetic miRNAs into zygotes partially conferred depression susceptibility to offspring and reshaped early embryonic transcriptional profiles [18]. Unfortunately, compared to recently developed technologies such as PANDORA-seq [56], our small RNA sequencing library, constructed using canonical methods, which would result in the omission of some end-modified non-coding RNAs from the results. Technological improvements to exploit the differences in sperm non-coding RNA will continue in the future. The failure to successfully recapitulate the infusions under PR scheduleinfusions under PR schedule increase observed in HSA-sired female F1 also leads us to consider the likelihood of other epigenetic factors affecting progeny and their potential interactions.

Another significant limitation is the need for further investigation into the specific molecular mechanisms by which miR-19b and other non-coding RNAs mediate the transgenerational inheritance of addiction-related behaviors. Multiple studies have found the involvement of miR-19 family in stress and memory consolidation, by modulating synaptic plasticity and receptor expression by targeting *Drebrin* and *Adrb1* [57, 58]. MiR-19b is also associated with many neurological diseases, including Alzheimer’s disease and Parkinson’s disease, through its interactions with key targets such as *Pten* and *Atxn1* [59–61]. Additionally, previous research has highlighted the key role of sperm miR-19b in the inheritance of metabolic adaptions [62] and transgenerational effects elicited by paternal voluntary exercise [63]. In our study, we identified miR-19b as one of the mediators in the epigenetic inheritance of heroin SA phenotype. Furthermore, we observed dysregulation of predicted miR-19b target genes in the NAc of HSA-sired F1 offspring, many of which are associated with drug addiction, such as like *Scn1a* [64], *Syt9* [65], *Creb5* [66], *Lepr* [67] and *Kcnj2* [68, 69], which warrants further experimental validations.

Notably, miR-19b can be generated from two precursors: miR-19b-1 on chromosome 15 and miR-19b-2 on chromosome X, which produce the same mature miR-19b [70]. Furthermore, estrogen can significantly downregulate miR-19b expression [71]. Since both precursors yield the same mature sequence, distinguishing between their sources is challenging. The dosage compensation mechanisms and escape from X-inactivation in females add complexity to how miR-19b-2 and other X-linked elements may differentially regulate gene expression and phenotype between sexes in addiction inheritance. Analyzing the epigenetic modifications of the promoters or regulatory elements of miR-19b-1 and -2 could provide insights into which alteration contributes more to the downregulation of miR-19b and enhance our understanding of sex differences in this context.

### Possible interaction mechanism of the brain with the sperm

Research has convincingly illustrated that paternal emotional perturbations [72], such as trauma [15–17], depression [18], environment enrichment [19], and drug addiction [11], can impact offspring behavior and cognition. Despite this, the exact pathway linking paternal mental states with the reproductive system remains elusive. In our correlational analyses of sperm non-coding RNA changes with the NAc, we observed a distinctive consistency in the changes of miRNAs, rather than other types of non-coding RNAs, between the brain and sperm, which underscores the critical role miRNAs play in the transgenerational transmission of psychogenic factors. This was supported by a few pioneering works. O’Brien et al. show that the artificial expression of human miR-941 in the dorsal striatum of the mouse was later detected in spermatozoa and fertilized eggs [73]. Furthermore, the Randal team also proved inter-tissue RNA or protein trafficking in modulation of reproductive biology using variable Cre-dependent expression in the brain [74]. These studies suggest that the consistent changes in miRNAs we observed between NAc and sperm are likely due to RNA transport rather than transcriptional regulation of miRNAs, which is a possible mechanism by which psychogenic factors affect sperm.

Non-coding RNAs in sperm undergo significant changes during spermatogenesis in the testes and maturation in the epididymis [75, 76]. This fluctuating profile of sperm non-coding RNAs not only coincides with the shedding of much of the cytoplasm during spermatogenesis, but also imply that environmental factors can exert influences at various stages of sperm development. This challenges the conventional wisdom that the window for modeling transgenerational inheritance must encompass the entire spermatogenic cycle. For instance, the miR-17-92 cluster, being present in testicular sperm but absent in spermatozoa of the epididymal head, only to reappear in the tail region [77], reflecting cytoplasmic shedding during spermatogenesis and environmental influences on sperm development. Current evidence suggests that cauda sperm are capable of rapidly responding to environmental cues, with significant changes in sperm non-coding RNA profiles observed as quickly as 7 days following a high-sugar diet in humans [78]. In line with this, our previous studies in rats have demonstrated that 7 days of paternal self-administration of sucrose [41] or a 14-day regimen of cocaine self-administration [11] can lead to altered offspring phenotypes. Moreover, through overexpression of miR-19b in the brain, we observed an upregulation of miR-19b in the sperm collected from the caudal epididymis after 14 days. These findings necessitate a reconsideration and further investigation into the temporal window within which paternal environmental factors can induce transgenerational epigenetic changes. It implies that shorter-term exposures could suffice to initiate heritable modifications, underscoring the remarkable sensitivity of the germline to environmental perturbations.

Additionally in the Supplementary Text (Figs. S9–S11), miR-19b overexpression in the NAc correlates with increased miR-19b in sperm from the caudal epididymis after 14 days, indicating that even short-term exposures to variable stimuli can lead to heritable changes, underscoring the germline’s sensitivity and rapid responses to environmental factors. And we provide some evidence of direct miRNA trafficking from the brain to the cauda epididymis sperm, as a possible explanation for the intriguing phenomena observed. The study of the effect of direct exchange between the nervous system and sperm offers the possibility of blocking the transgenerational inheritance of drug addiction in the future.

## Supplementary information

Raw data and statistics

Primer sequences used

Supplementary text, experimental procedure, and figures

R code for statistics and plotting

## Data Availability

All data are available in the supplementary materials. Raw NGS data are deposited in the NCBI BioProject database under accession number PRJNA780605.
