# Supplementary material for: Paternal heroin self-administration in rats increases drug-seeking behavior in male offspring via miR-19b downregulation in the nucleus accumbens
Source: Neuropsychopharmacology. 2025 Mar 8;50(7):1027–38. doi: 10.1038/s41386-025-02081-8 (PMC12089436; doi:10.1038/s41386-025-02081-8)
Supplement: Supplementary file 3 — Supplementary text, experimental procedure, and figures [file 41386_2025_2081_MOESM3_ESM.pdf]

Supplementary Materials for

Paternal Heroin Self-Administration in Rats Increases Drug-Seeking Behavior in Male Offspring via miR-19b Downregulation in the Nucleus Accumbens

This PDF file includes:

Supplementary results and discussions

Experimental procedures for experiments 1 to 18

References

Figures S1 to S11

Supplementary results and discussions

Concordant brain-sperm miR-19b changes is possibly mediated by RNA trafficking

Consistent changes of miRNA in the NAc with the sperm may implicate a potential mechanism through which heritable changes may transmit from the brain to the sperm. We then investigated miR-19b as a proxy. Synthetic miR-19b structured within a miR-30 stem-loop was subcloned into an AAV vector, thereby enabling Cre-driven expression of synthetic miR-19b (see Figures S6a–d). Subsequently, we delivered the packaged AAV into the NAc in conjunction with hSyn-Cre to ascertain the level of miR-19b in the NAc, testis, caput epididymis, and cauda epididymis 1, 2, and 4 weeks after (Figure S9a). With the elevation of miR-19b expression in the nucleus accumbens (Figure S9b) ($F_{group×time}(2, 27) = 5.94$, $P = 0.00729$), miR-19b levels exerted a surprisingly significant up-regulation in spermatozoa of the cauda epididymis (Figure S7c, $F_{group×time}(2, 27) = 6.36$, $P = 0.00546$), but not in the testis (Figure S9c, $F_{group×time}(2, 27) = 0.739$, $P = 0.487$) or caput epididymis (Figure S9c, $F_{group×time}(2, 27) = 0.441$, $P = 0.648$). A significant positive correlation was observed between NAc and miR-19b levels in cauda epididymis sperm (R = 0.656, $^{**}P = 0.00578$), with no correlation found in other tissues (Figure S9d, testis, R = 0.077, $P = 0.777$; caput epididymis, R = 0.265, $P = 0.321$). Four weeks after injection, NAc miR-19b levels increased ~100-fold (Figure S9e, $t(3) = 3.64$, $^{*}P = 0.0356$), though only slight increases were noted in the mPFC ($t(5.40) = 2.73$, $^{*}P = 0.0382$), and no changes were observed in peripheral tissues like the liver($t(3.09) = -0.0222$, $P = 0.984$), lymph nodes ($t(3.66) = -0.864$, $P = 0.440$), spleen ($t(4.54) = 0.400$, $P = 0.707$), or blood ($t(5.95) = -0.0255$, $P = 0.981$) (Figure S9e). These results indicate that upregulation of miR-19b in the NAc

significantly affects mature sperm levels in the cauda epididymis but not during spermatogenesis in the testis or caput epididymis.

We first considered the possibility that NAc miR-19b upregulation could activate miR-19b transcription in other tissues. In this way, upregulation of miR-19b precursor could be found. However, quantitative PCR targeting the endogenous precursor of miR-19b revealed no such upregulation in all tissues we assayed (Figure S10a-b, liver, $t(4.37) = 1.64$, $P = 0.171$; lymph node, $t(3.68) = -0.406$, $P = 0.707$; spleen, $t(4.20) = 0.425$, $P = 0.692$; blood, $t(5.71) = 0.637$, $P = 0.548$; NAc, $t(4.17) = -0.835$, $P = 0.449$; mPFC, $t(5.51) = -1.14$, $P = 0.3027$). Additionally, we excluded potential effects of AAV leakage. PCR targeting pre-miR-19b from the viral vector showed no significant changes in peripheral tissues, while elevated levels were confirmed in the NAc ($t(3) = 5.33$, $*P = 0.0129$) and mPFC ($t(3) = 4.23$, $*P = 0.024$, Figure S10c). Copy number quantification of viral DNA components showed detectable viral particles only in the NAc (CMV, $t(5) = 3.24$, $*P = 0.0228$; hSyn $t(5) = 2.50$, $P = 0.0548$; Cre, $t(5) = 2.54$, $P = 0.0516$), and adjacent nuclei like dStr (CMV, $t(5) = 2.67$, $*P = 0.0444$) and mPFC (hSyn, $t(5) = 2.74$, $*P = 0.0408$), absent in peripheral tissues (Figure S10d-f). These findings suggest that the rise in miR-19b levels in cauda epididymis sperm is likely due to direct transport rather than local transcription or viral leakage.

Given the specificity of miR-19b changes in the cauda epididymis, we hypothesized that dynamics of non-coding RNAs in the cauda epididymis arise from vesicle exchange. To investigate this, we administered the noncompetitive neutral sphingomyelinase (N-SMase)

inhibitor GW4869 into the lateral ventricle for four days, collecting tissues for PCR on the fifth day (Figure S11a). MiR-19b levels were significantly downregulated in the cauda epididymis ($t(13.9) = -7.42$, $P = 3.36e-6$), but unchanged in other tissues (NAc, $t(13.0) = -1.44$, $P = 0.173$; testis ($t(7.62) = -0.0712$, $P = 0.945$; caput epididymis, $t(8.87) = 0.58$, $P = 0.576$), indicating that extracellular vesicle release from the brain regions including the NAc may mediate miRNA changes in cauda epididymal sperm (Figure S11b). Furthermore, analyses of miR-19b levels in extracellular vesicles from the NAc, plasma, testis, and both epididymal regions after viral overexpression showed elevated levels in NAc and cauda epididymis vesicles (Figure S11c-d, NAc EV, $F_{group×time}$ (2, 27) = 4.25, $P = 0.0248$; plasma EV, $F_{group×time}$ (2, 27) = 1.01, $P = 0.378$; testis EV, $F_{group×time}$ (2, 27) = 0.004, $P = 0.996$; caput epididymis EV, $F_{group×time}$ (2, 27) = 0.72, $P = 0.496$; cauda epididymis EV, $F_{group×time}$ (2, 27) = 9.78, $P = 0.000637$), significantly correlating with cauda epididymis EV miR-19b levels (Figure S11e, miR-19b overexpression, NAc EV *vs.* cauda epididymis EV, R = 0.657, $P = 0.005$). These data suggest that extracellular vesicles play a crucial role in transporting miR-19b from the brain to spermatozoa.

Our data supports extracellular vesicles (EVs) transporting miRNAs from the brain regions, including the nucleus accumbens to sperm, as inhibition of SMNase activity can lead to downregulation of sperm miR-19b. This is supported by observations where human miR-941, initially artificially expressed in the mouse dorsal striatum, was later detected in sperm and fertilized eggs[1]. EVs facilitate communication within the CNS and between the CNS and peripheral systems[2,3]. In the reproductive system, epididymal epithelial cell-derived EVs deliver non-coding RNAs to immature sperm[4]. Notably, no changes in blood levels of EV-

carried miR-19b were observed, possibly due to blood EV heterogeneity or alternative trafficking routes. We also extracted cerebrospinal fluid for testing (data not shown), but no significant changes in EV miR-19b levels were seen there either. These results argue against a somatic EV delivery pathway.

Anatomical studies have shown that the head and tail of epididymis is differentially innervated by sympathetic, parasympathetic, and motor nerves. While retrograde neuronal tracings revealing connections to the brainstem in both segments of the epididymis[5]. While the function of the neurons were largely unclear, sympathetic denervation of the cauda epididymidis changed luminal fluid protein composition[6], and partial denervation of the testis imply that local opioid actions are modulated by testicular nerves[7]. These findings collectively hint at a neural-EV axis as a pivotal means by which psychogenic factors may impart reproductive events. Recent studies revealed that EVs can ferry proteins between distant brain areas[8], and surprisingly on neuron surface[9], implying neurons as a conduit for EV transit. Uncovering the intricacies of this pathway stands as a crucial frontier in understanding and mitigating intergenerational transmission of mental and behavioral traits influenced by paternal experiences.

Experimental procedures for experiments 1 to 18

Experiment 1 Heroin Self-administration of naïve SD rats to generate F1 and F2 for behavioral assessments

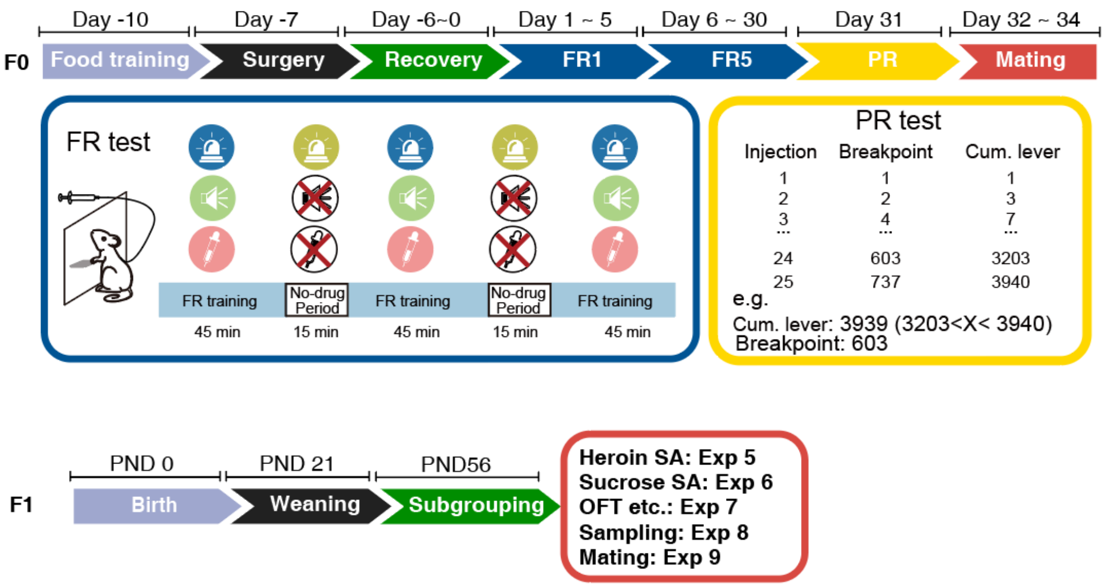

Figures involved: Figure S1a-c

Procedure:

The F0 generation rats were all male Sprague-Dawley rats purchased from the Shanghai Laboratory Animal Center, Chinese Academy of Sciences. They were acclimated to the facility for a least one week prior to the start of each procedure until they reached 8 weeks of age. Before self-administration, rats were food-restricted to maintain a body weight of 85% of their ad libitum body weight and were then trained to press the active lever to obtain a food pellet (45 mg, Bio-Serv, USA) in operant chambers (Med Associates Inc., USA). Each operant chamber was equipped with two levers, a house light (to signal drug/no-drug periods), a blue cue light (to signal lever availability) and a white cue light (to signal drug infusions), and a loudspeaker. The food training procedure was conducted over a five-day

period, with each day consisting of a four-hour training session. The entire training procedure consisted of three regimens: the first 20 pellets are obtained by fixed-ratio one (FR1) lever pressing with a 4-second cue (light and tone) and no time-out, then 30 pellets is obtained by FR1 lever pressing with a 20-second cue and a 20-second time-out period, and the last 50 pellets is obtained under FR5 with a 20-second cue and a 20-second time-out period. Rats were usually able to successfully complete the training procedure within three sessions, obtaining a total of 100 food pellets.

The rats were anaesthetized with isoflurane (2.5%) and a chronic indwelling jugular catheter (ID = 0.31 mm, OD = 0.64 mm, Dow Inc., USA) was implanted into the right jugular vein and connected to a homemade back-mounted pedestal. Rats were allowed to recover for seven days prior to the start of behavioral testing. For the first three days after catheter implantation, lincomycin (0.5%) and lidocaine (0.4%) gel (Shanghai Pharmaceuticals, Shanghai, PRC) was applied to the incisions on the back and neck. In addition, the catheters were flushed daily with 0.1 mL of saline containing heparin (30 $IU \cdot ml^{-1}$) and gentamycin (0.5 $mg \cdot ml^{-1}$) to maintain catheter patency.

Following the recovery phase, rats were subjected to self-administration of heroin. The dose of heroin administered was 45 $\mu g \cdot kg^{-1}$ per injection, delivered over a period of four seconds. The experimental paradigm employed a 5-day fixed ratio of 1 (FR1), 25-day FR5, and 1-day progressive ratio (PR) tests. In a single batch of animals (Experiment 12), the experimental paradigm was modified to consist of a 5-day fixed ratio of 1 (FR1), a 10-day FR5, a 1-day PR, a 15-day FR5, and a 1-day PR.

In the context of a fixed-ratio schedule, rats were trained to press the lever over the course of a daily 2.5-hour session. This comprised three 40-minute drug-available periods, interspersed with two 15-minute drug-unavailable periods, which were referred to as "no-drug periods". The availability of the drug was indicated by the illumination of a blue cue light, whereas the absence of the drug was indicated by the illumination of the chamber light and the extinction of the blue cue light. When rats pressed an active lever a preset number of times, a drug infusion was delivered over 4s, and the white cue light was turned on for 20 s accompanied by a tone cue. Each infusion was followed by a 20 s time-out period during which further lever presses were recorded but did not result in additional food or drug dispenses.

Rats were tested on a progressive ratio schedule paradigm to assess their motivation for reinforcement. The lever press requirements for each injection (i) follow the equation i^{th} injection=Int ($5e^{0.25i-5}$) and the session stops when rats take more than 1 h to satisfy the response requirements, or the session was terminated 2.5-h. The break point, i.e., the lever press required for the last injection, was recorded and used to assess reinforcer-seeking motivation for each rat.

A total of 7 batch of naïve rats were tested. The performance of each individual rat behavioral score (X) was calculated by its lever presses during FR5 reinforcement schedule and break point during PR reinforcement schedule, following $X = \frac{(X_i - \bar{X})}{s.d.}$. X_i represents the value of behavior performance of each rat, $\bar{X}$ is the mean behavior value for all the rats of the same batch, and s.d. is the standard deviation of the batch of animals tested. Saline F0 generation were randomly selected from rats that self-administered saline. The rats randomly chosen from the top 40% of

additive score served as HSA F0 generation except in experiment 12, we mated one whole batch of HSA rats to generate F1. Twenty-four hours after the last self-administration session, the designated F0 were respectively housed with two naïve female rats to generate F1 generation, and removed after 4 days.

For the F1 generated, the litter size was reduced to a maximum of 10 at PND 10, with 5 male and 5 female F1 offspring. After weaning, each single sex litter was kept in a cage.

Offspring were bred in the laboratory under clean animal husbandry (CL) conditions. They were subjected to heroin self-administration tests (Experiment 5), sucrose self-administration tests (Experiment 6), open field, LDB, EPM, Y and social tests (Experiment 7), used for sampling (Experiment 8) or bred to generate F2 (Experiment 9). Two to three rats of each sex were randomly selected from each litter and used for each experiment. Rats used for different purposes were not mixed.

Experiment 2 Self-administration and yoke infusion of heroin in naïve SD rats to generate F1, along with the collection of biological samples

Experimental design:

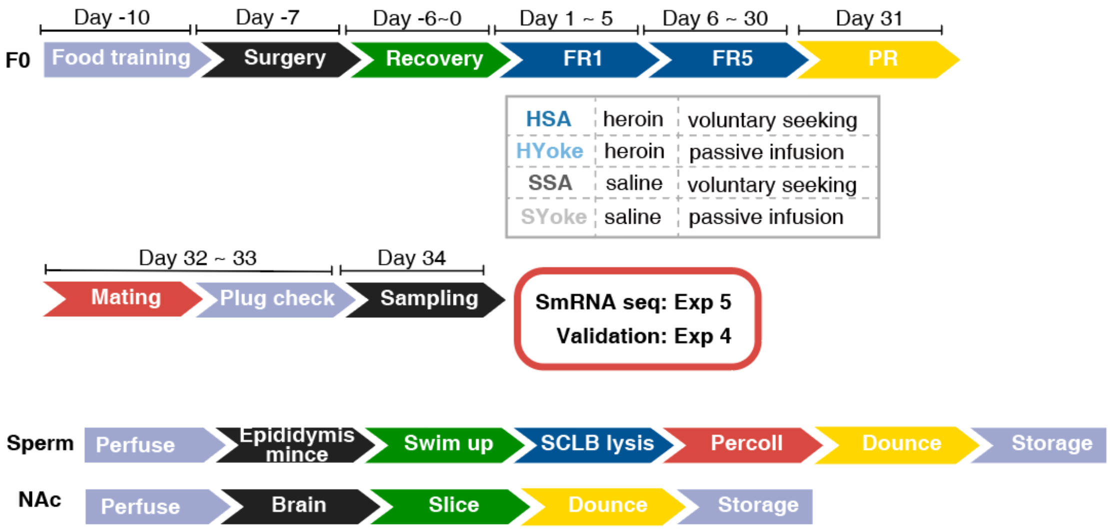

Figures involved: Figure 2a

Procedure:

The food training, surgery, recovery and heroin self-administration procedures were the same as in Experiment 1, except that after surgery, rats were randomly assigned to perform voluntary self-administration or to receive yoked infusions. During the self-administration procedures, two rats were randomly paired; one rat could freely press the lever for the injection of heroin or saline, while the other (yoked) rats passively received drug injections at the same dose, time and rate. For yoked rats, pressing the active lever resulted in no drug injection or conditioned cue.

Rats were housed with two naive female rats for 24 hours after the last self-administration. Copulation was checked 6 hours after. The plugged female rats were then housed individually until delivery. The litter size was reduced to a maximum of 10 at PND 10, with 5 male and 5

female F1 offspring. After weaning, each single sex litter was kept in a cage. Offspring were bred in the laboratory under clean animal husbandry (CL) conditions. They were subjected to heroin self-administration tests (Experiment 5), heroin dose-response tests (Experiment 9), or sampling (Experiment 4). Two to three rats of each sex were randomly selected from each litter and used for each experiment. Rats used for different purposes were not mixed.

Male rats that successfully copulated with the female were sacrificed as soon as plugging was observed in female rats. They were euthanized with an overdose of isoflurane, after which the thoracic cavity was opened in order to expose the heart. The right auricle of the rat was then clipped, and 50 mL of ice-cold saline was perfused into the left ventricle with an 18-gauge syringe needle. Subsequently, the brain, the bilateral epididymis and vas deferens were removed and dissected on ice for sampling.

In the case of brain samples, the entire brain was placed within a brain matrix and sliced into 1.0 mm slices using a sharp blade. The regions of interest (NAc, mPFC) were then rapidly dissected and Dounce homogenized in Trizol reagent (Vazyme Biotech). These samples were stored at -20°C until RNA isolation.

Adipose and connective tissues were carefully removed from the epididymis. The caudal part (1/3 connected to the vas deferens) was removed and rinsed twice with saline solution. Cauda epididymal spermatozoa were collected by squeezing the vas deferens and making incisions at both ends of the tissue and flushing the seminiferous tubules with 37°C pre-warmed saline using a 26-gauge needle. The rinsed cauda epididymis was further minced and incubated in a total of 3 mL of saline at 37°C for 10 min, and the top 2 mL of supernatant was transferred to a new

conical tube, lysed in somatic cell lysis buffer (0.1% SDS, 0.5% Triton X-100 in nuclease-free H_2O) on ice for 30 min, followed by centrifugation at 2,000 g for 5 min at 4°C. The pellet containing spermatozoa was then resuspended in HBSS buffer, and gently layered on top of a 35% (v/v) Percoll solution prepared in HBSS. The tubes were then centrifuged at 1000 × g for 8 minutes without brake to ensure minimal disturbance of the gradient. Then the pellet at the bottom of the tube, enriched for pure spermatozoa, was homogenized in Trizol reagent and stored at -20°C until RNA isolation.

Two separate batches of tissue samples were taken. One was used for small RNA sequencing (experiment 3) and RNA injection (experiment 13), and the other for validation of candidate miRNA expression (experiment 4).

Experiment 3 Small RNA-seq and subsequent analysis

Figures involved: Figures 4a-e, Figure S3

Procedure:

Trizol-lysed samples from Experiment 2 were then used for RNA extraction. For sperm samples, the homogenate was allowed to warm to room temperature, and then subjected to two cycles of heating at 65°C for 5 minutes-dounce homogenizations to ensure complete lysis of the samples. The heated sperm homogenates and brain samples were then centrifuged at 13,200g for 5 minutes to remove debris. RNA was then extracted using the Direct-zol® RNA Microprep Kit (Zymo Research) with slight modifications. The supernatant was collected, mixed 1:1 with absolute ethanol, transferred to the column and centrifuged. The column was then incubated with 5 U RNase-free DNAse I (NEB) in DNA digestion buffer for 15 min at room temperature and washed with RNA pre-wash and RNA wash buffer. Finally, RNA was eluted with 65°C nuclease-free water (Ambion) and quantified using the RNA High Sensitivity Kit on the Qubit 3.0 Fluorometer (Thermo Fischer Scientific).

For sequencing and microinjection samples, the RNA integrity number (RIN) was further determined using 1 ng RNA on the Agilent 2100 Bioanalyzer with the Agilent RNA 6000 Pico Kit (Agilent Technologies) according to the manufacturer's instructions. Samples from each rat with brain RNA RIN $\geqslant$ 8 and sperm RNA with ribosomal contamination of below 0.5% were included.

For small RNA library preparation, 200 ng of total RNA was subjected to library construction using the VAHTS^{TM} Small RNA Library Prep Kit for Illumina (Vazyme LLC) according to the

manufacturer's recommendations for RNA denaturation, adapter ligation, cDNA synthesis, and library amplification. The PCR product was then purified using 1.8 × DNA Clean Beads (Vazyme LLC), and then loaded on 6% non-denaturing TBE acrylamide gel for size selection. Bands ranging from 140 bp to 190 bp was recovered by crushing and soaking in acrylamide gel elution buffer (0.5 M NH_4Ac_3, 1 mM EDTA pH8.0, DEPC treated) at 37°C on a rotary platform for 3-4 h[10]. DNA was then recovered using the Zymo DNA Clean & Concentrator-5 kit (Zymo Research), quantified using the P5-Q and P7-Q primer pairs (see Supplementary Table 2) and pooled. Sequencing was performed on Illumina MiSeq (75 bp single end, SE) or NovaSeq 6000 (150 bp paired end, PE, by pooling with other transcriptomic samples) by Azenta Life Science with an average sequencing depth of 10 million reads per sample.

Raw small RNA sequencing data was first processed using NGmerge[11] to merge paired-end reads and remove sequencing adapters. The assembled sequences were then analyzed using Sports1.0[12]. Bowtie[5] indexes for annotation were generated using the following versions of rat reference databases: miRNA, miRbase V21.0[13,14]; mRNA, rRNA, GtRNA, mitotRNA, piRNA were masked from Ensembl rn6.0; tsRNA from GtRNAdb using Rat Jul. 2014 RGSC 6.0[15].

The counts for each non-coding RNA was subjected to differential expression analyses using the R package DESeq2[16] under default settings. Significance was set at an adjusted p-value ≤ 0.05. Heatmaps were plotted using log-transformed and variation-stabilized counts with R package "pheatmap". The concordance of microRNA expression signature was evaluated using RRHO2 package [17]. The tissue specificity of miRNA was analyzed using miEAA2.0[18].

For prediction of key miRNAs that regulates addiction-related process, a network propagation-

based model was generated as described[19]. Briefly, miRNA families and the potential targets that are highly conserved (conservation score ≥ 2) from TargetScan Human 7.1 were used. Potential addiction-related genes, as well as their corresponding reliability score, were from Li et al.[20], log-transformed and used as candidate lists for correlation calculation with the algorism. NPES score for each microRNA was extracted for miRNA ranking[21].

Experiment 4 Validation of miRNA expression in F0 generation

Figures involved: Figures 4f-g

Procedure:

For microRNA expression validation, samples from Experiment 2 were used. RNA was reverse transcribed using protocol adapted from S-Poly(T) Plus method[22]. Briefly, 10 μL of reverse transcription mix, made up of 100 ng total RNA, 1 μL of primer panel containing 0.5 μM of each miRNA RT primer (see Supplementary Table 2), 0.8 U poly-A polymerase (New England Biolabs), 100 U Superscript II reverse transcriptase (Thermo Fisher Scientific), 25 mM Tris pH 8.0, 75 mM NaCl, 5 mM $MgCl_2$, 500 μM ATP, 250 μM dNTP, heated at 25°C for 30 min, 42°C for 30 min, and 65°C for 5 min.

Real-time quantitative PCR was carried out with 10 μL reaction mix containing 0.4 μL RT product, 100 nM of forward primer and miRNA-specific primer (see Supplementary Table 2), 5 μL 2 × ChamQ Universal SYBR qPCR Master Mix (Vazyme Biotehc). PCR was carried out on CFX Opus 384 (Bio-Rad), 95°C for 5 minutes, followed by 40 cycles at 95°C for 10 s, and then 60°C for 30 s. U6 was used as internal control. All reactions were run in triplicate. Samples with between-sample cycle threshold (CT) variation ⩾ 0.3 were re-assayed. Then ΔΔCT method was used to quantify relative gene expression. First, the ΔCT is calculated by subtracting the reference gene's CT from the target gene's CT within each sample to normalize expression levels. Then, to calculate between-group variation, the ΔΔCT is derived by subtracting the averaged ΔCT of control group (SSA-F0) from the ΔCT of each experimental sample. Relative gene expression is then calculated as $2^{\wedge(-\Delta\Delta CT)}$, assuming 100% PCR efficiency.

Experiment 5 Heroin self-administration tests of F1 offspring

Figures involved: Figures 1a-c Figures 2a-b

Procedure:

For F1 generation heroin self-administration tests, 8-week-old rats, 2-3 from each litter sired by HSA F0, SSA F0, HYoke F0, SYoke F0, were subjected to food training, surgery, recovery as described in Experiment 1 and tested in a 3-day FR1, 6-day FR5, 1-day PR paradigm. Number of lever presses during drug-available periods of FR sessions, drug infusions per session, were recorded. While break point under the PR paradigm, i.e. the lever press required for the last injection, was recorded and used to assess reinforcer-seeking motivation for each rat.

The experimenter was blind to the rat's group until the end of the experiment. Rats with a blocked catheter or severe post-operative infection were excluded from data collection. If instrumental problems occurred (rat chewed or removed the infusion tube, lever or lamp failure), the data for that day were deleted.

Experiment 6 Correlation analysis of F0 and F1 on heroin-seeking behavior

Figures involved: Figure S1d-m

Procedure:

A cohort of 16 naïve male SD rats were subjected to food training, surgery, and recovery as described in Experiment 1. Then they were all subjected to heroin self-administration training according to an adapted paradigm: 5-day FR1, 10-day FR5, PR test-1, 15-day FR5, and PR test-2. The lever-pressing performance of each rat under fixed-ratio reinforcement program, no-drug periods, as well as in PR tests were recorded. Then, they were all subjected to mating to generate

F1. Male F1 offspring were again tested for heroin self-administration tests with 3-day FR1, 6-day FR5, and one PR test. The behavior of F0 and F1 were z-scored and compared.

Experiment 7 Sucrose self-administration tests of F1 offspring

Figures involved: Figures 1d-e

Procedure:

F1 generation rats, with *ad libitum* food access, were trained to press the lever to obtain sucrose pellets (45 mg, Bio-Serv, USA) in daily 1 h sessions using the FR1 reinforcement schedule for 5 sessions, then the FR5 reinforcement schedule for 5 sessions, and the FR10 reinforcement schedule for 5 sessions. Rats were then tested using the PR schedule (2.5 h) for 1 session. When rats pressed an active lever a pre-determined number of times, a sucrose pellet was delivered into a magazine and the white cue light was turned on for 20 s accompanied by a tone cue. Each pellet delivery was followed by a 20-s time-out period during which further lever presses were recorded but did not result in additional responses, and rats that failed to acquire 10 pellets in the last FR10 session were excluded from the analysis.

Rats were tested using a progressive ratio schedule paradigm to assess their motivation for reinforcement. Lever-press requirements for each pellet delivery (i) followed the equation i^{th} pellet=Int ($5e^{0.25i-5}$) and the session was terminated if rats took more than 1 h to meet the response requirements, or the session was terminated after 2.5 h. The break point, i.e. the lever press required for the last injection, was recorded and used to assess reinforcer-seeking motivation for each rat.

Experiment 8 Behavioral tests on anxiety, short-term memory and sociability of F1 offspring

Figures involved: Figure S2

Procedure:

A separate batch of eight-week-old rats was used for a series of behavioral tests. Three days prior to the start of all tests, the rats were acclimated to the test room for 1 hour per day. The order in which the tests were conducted was as follows: open field test, elevated plus maze test, light/dark box test, sociality test and Y-maze test. In each day one single test was performed, with a minimum interval of one day between each test. On each day, rats were acclimated to the test room for at least 1 h prior to testing. Tests started at the 14:00 ZT (Zeitgeber time, two hours after the extinction of lights) and ended before the 22:00 ZT. The experimenter was blind to the group of the rats tested.

Open field test Locomotor activity was measured in open-field activity chambers (MED Associates Inc., USA, 43 cm × 43 cm × 30 cm) for rats. The light intensity in the center of the arena was adjusted to 60 lux. Rats were placed in the chamber at the commencement of the test, facing the wall. Distance travelled, time spent in the central area (30 cm × 30 cm), and entries into the center were recorded for 30 min. At the conclusion of each test, the chambers were sanitized using 75% ethanol to eradicate any olfactory cues.

Elevated plus maze test The apparatus was elevated 50 cm above the floor and consisted of two closed arms (30 cm long, 30 cm high, and 10 cm wide), two open arms (30 cm long and 10 cm wide), and an exposed central panel (10 cm × 10 cm). The rat was placed in the center of the maze, facing the closed arm. The field was illuminated to a level of 50 lux, ensuring minimal

shadowing in the closed arms. The session was recorded by a top-viewed video camera for 6 min, and the time spent in the open arm was calculated using Clever System software (CleverSys. Inc., USA). It should be noted that time spent in the middle was excluded from the calculations. The apparatus was wiped with 75% ethanol to remove any olfactory cues after each test rat.

Light-dark box test The apparatus for the light-dark box test comprised one dark chamber and one transparent chamber of equal area (MED Associates Inc., 40 cm × 20 cm × 30 cm each), separated by a wall with an opening at the bottom to allow unobstructed passage of the rats. The field was illuminated by an incandescent lamp, with the result that the brightness of the light side of the box averaged 65 lux. The rats were positioned within the opaque chamber, and the test commenced when the door to the illuminated chamber was raised. The time and distance traversed by the rats within the apparatus were measured for a period of 15 minutes using the Med Associates activity monitor program. Following each trial, the apparatus was cleansed with 75% ethanol to eliminate any olfactory cues.

Three-chamber sociability and social recognition test The apparatus was constructed of clear Plexiglas and consisted of two end chambers (60 cm × 40 cm × 50 cm) and a central chamber (30 cm × 40 cm × 50 cm) separated by arched doors. Prior to testing, the rats used as stranger rats were habituated to the restraint cage (O.D. 20 cm; height, 30 cm) for 10 minutes, once a day for 3 days. The test rat was allowed to freely explore the apparatus for 10 minutes the day before the test. On the day of the test, two test phases were performed. In the sociability test, an empty restraint cage and a restraint cage containing an unfamiliar rat (stranger A) were

placed in the two end chambers. The social recognition test was conducted by allowing the test rat to explore freely for 10 minutes while being video recorded. Then, to assess social recognition ability, another age- and sex-matched control rat (Stranger B) was placed in the empty cage and Stranger A was placed as before. The test rat was again allowed to freely explore the chamber for a further 10 minutes. The time spent by the test rat sniffing and exploring the empty cage or interacting with either Stranger A or B was manually quantified by an investigator blinded to the identity of the test rat. Chambers used to house the empty cage or the cage containing the unfamiliar rat were randomized across trials.

Y-maze The apparatus was constructed of beige Plexiglas and contained three arms (30 cm long, 30 cm high, and 10 cm wide) at 120 degrees to each other. The three arms were randomly designated as the familiar arm, the start arm, and the test arm for each individual rat. During training, the test rat was placed in the start arm and allowed to explore the maze for 10 minutes with the test arm blocked. One hour later, all arms were made available and the test rat was allowed to explore the maze for 5 minutes while being videotaped. After each test, the apparatus was wiped with 75% ethanol to remove any olfactory cues. Time spent in each arm was manually quantified by an investigator blinded to the identity of the test rat. Novelty preference was calculated as $\frac{\text{time spent in novel arm}}{\text{time spent in all arms}} \times 100\%$.

Experiment 9 Dose-response tests of F1 offspring

Figures involved: Figure 2c-d

Procedure:

A separate pool of F1 rats was used for dose-response curve testing. They underwent food training, surgery and initial fixed-ratio training as in Experiment 5, and were then trained to lever-press to obtain a variable dose of heroin (67.5, 45, 27, 20, 6.75, 4.5, 2, 0 $\mu g\text{-}kg^{-1}$, intravenously) in descending order under the FR5 reinforcement schedule. Again, a daily 2.5-hour training session consisted of three 40-minute drug-available periods interspersed with two 15-minute drug-free periods. Each dose was maintained for 3-5 days and until the rats' response was stable over 2 days (< 10% variation), and then switched to the original training dose (45 $\mu g \cdot kg^{-1}$) until the rats' response was stable over 2 days (< 10% variation), and then switched to the next dose tested. Mean lever press was calculated from the lever pressed during the last 2 days of each dose.

Experiment 10 Generation and heroin self-administration tests of F2 offspring

Experimental design:

Figures involved: Figure 1f-g

Procedure:

F2 generations were generated by crossing naïve male F1 generation from each litter with two naïve female rats. Eight-week-old rats, 2-3 from each litter sired by HSA F1, SSA F1were subjected to food training, surgery, recovery as described in Experiment 1 and tested in a 3-day FR1, 6-day FR5, 1-day PR paradigm. Number of lever presses during drug-available periods of FR sessions, drug infusions per session, were recorded. While break point under the PR paradigm, i.e. the lever press required for the last injection, was recorded and used to assess

reinforcer-seeking motivation for each rat.

The experimenter was blind to the rat's group until the end of the experiment. Rats with a blocked catheter or severe post-operative infection were excluded from data collection. If instrumental problems occurred (rat chewed or removed the infusion tube, lever or lamp failure), the data for that day were deleted.

Experiment 11 Transcriptomic sequencing of F1 offspring

Experimental design:

Figures involved: Figure S4a-c, Figure 5c-d

Procedure:

We proceeded to sequence the transcriptome of the NAc in a series of experiments. In the first series, we examined naïve female and male SSA-sired F1 rats, HSA-sired F1 rats, as well as HYoke-sired F1 rats. In a second series, the NAc of the HSA-sired male F1 rats were transfected with miR-19b. This series of experiments was undertaken in order to detect the effect of transiently overexpressing microRNA on transcript levels in F1 generation NAc tissues. In both series, two rats from the same litter were mixed at the same molar ratio to produce one sample. For transfection, Double-stranded agomir mimicking mature miR-19b or random control sequences (purchased from Genepharma, see Supplementary Table 1) were dissolved in Phosphate Buffered Saline (PBS) to a concentration of 2,000 ng·ul^{-1} and mixed with DOTAP Liposomal Transfection Reagent (Roche Diagnostics GmbH) in a 1:4 ratio The mixture was then left to stand at room temperature for 10 minutes, after which bilateral injections were made

into the NAc. Briefly, rats were anaesthetized with isoflurane (2.5%) and placed in a stereotaxic instrument (RWD Life Science). The skin on the top of the head was cut with a sharp scalpel to expose the skull, and the incisor bar was adjusted until the lambda and bregma heights were equal. The skull was then drilled at the coordinate AP: 2.0 mm; ML: ± 1.2 mm above the dura mater. All infusions were performed using glass pipettes with Nanoject III (Drummond Scientific Co., USA) and delivered at 0.24 $\mu L \cdot min^{-1}$ in 5 minutes on each side (DV: -7.0 mm). After each injection, the injection needle was left in the brain for 10 min to allow sufficient diffusion. The skin was then sutured closed.

Five days after the transfection, the rats were euthanized and the NAc was harvested and subjected to RNA extraction (as in Experiment 2). A total of 100 ng RNA was reverse transcribed, and used for real-time fluorescence quantitative PCR analysis of miR-19b expression. Then 300ng total RNA from NAc was then used for transcriptomic sequencing.

The assessment of the concentration and quality of the purified RNA was conducted as in Experiment 2. Then construction of the transcriptomic library was done using the Ribo-off rRNA Depletion Kit and VAHTS Universal V8 RNA-seq Library Prep Kit for Illumina (Vazyme), according to manufacturer's protocol. Total RNA was incubated with rRNA probes to form rRNA-probe hybridization complexes, followed by the degradation of these complexes using RNase H. Subsequent to this, the remaining probes were removed by DNase I, and the RNA was fragmented. Universal libraries are obtained by adding dNTP to complementary DNA synthesis and ligating to RNA sequencing junctions, and libraries of insert fragments in the range of 200 ~ 450 bp are enriched by magnetic bead screening. Strand-specific libraries are

obtained by adding dUTP to complementary DNA synthesis and ligating it to the RNA junction, and insertion fragment libraries in the range of 150 ~ 550 bp are enriched by magnetic bead screening. The sequencing process was conducted using a Hiseq 3000 instrument, yielding an average depth of 50 million reads.

For the total RNA libraries, low-quality (Phred score < 28) and too-short reads (< 30 bp) were filtered using Trimmomatic (v0.32)[23]. Subsequent to the cleansing of the data, the reads were mapped to rat genome (Ensembl Rnor_6.0) with Hisat 2 (v2.0.2) [24]. Annotation and quantitation were subsequently conducted using featureCounts from Subread (v1.6.2)[25]. The differential analysis of genes was performed using DESeq2 (v1.26.0)[16]. Genes that were expressed in two-thirds of all samples, with a minimum of 80 counts and a P value of $\leqslant$ 0.05, were considered to be significantly differentially expressed. Gene ontology annotation, overrepresentation analysis, and geneset expression analysis were done using R package clusterProfiler[26]. To examine the transcriptome profiles of “Predicted targets of core miRNAs”,predicted targets of the 24 miRNAs from Figure 4e were extracted from TargetScanMouse (V8.0)[27], combined and used as the candidate gene set. GO enrichment network was constructed using ClueGO and Cluepedia[28] using Cytoscape (v3.7.2).

Experiment 12 MiRNA qPCR of the nucleus accumbens of F1 offspring

Figures involved: Figure S4d-e

Procedure:

NAc from naive female and male F1 rats from the SSA, HSA and HYoke groups were used for

RNA extraction (the same sample as the first series of Experiment 11). RNA from two rats from the same litter was mixed in the same molar ratio to produce one sample. The RNA was then reverse transcribed and used for qPCR analysis of miRNA levels according to Experiment 4.

Experiment 13 Validation of AAV-based miR-19b overexpression, and behavioral assessment of miR-19b overexpression of heroin self-administration in NAc of HSA-sired F1

Figures involved: Figures S7a-d, Figure 5e

Procedure:

Cre-dependent miRNA overexpression was achieved by cloning synthetic miR-30-based scaffold overexpressing miR-19b (AGTTTTGCAGATTTGCAGTTCAG) or scramble sequence (GAGCAATTATTGTGTTGCCGTT) into pAKD-CMV-bGlobin-Flex-EGFP-MIR30shRNA vector using EcoRI/XhoI restriction sites[29]. Successful cloning of MIR30-miR19b was validated using Sanger sequencing. Then the plasmid was prepared using ZymoPURE™ II Plasmid Maxiprep Kit (Zymo Research), and used for transfection validation and AAV preparation.

For validation of miR-19b over expression in vitro, HEK293T cells were purchased from the National Collection of Authenticated Cell Cultures (Shanghai, China), and cultured in Dulbecco’s modified Eagle’s medium containing 10% FBS. Cells were seeded 18-20 h before transfection and transfected with calcium phosphate/DNA co-precipitation method. For plasmid expression validation, cell was transfected with pCDNA3.0-CAG-Cre, together with AAV2/9-flex-miR19b-EGFP or with AAV2/9-flex-Scr-EGFP, and RNA was extracted from harvested cell 36 hr post transfection. The procedure of RNA extraction and reverse transcription can be

found in Experiment 2 and 4.

AAV2/9-flex-miR19b-EGFP, AAV2/9-flex-Scramble-EGFP were prepared in the library. Briefly, the constructed backbone was co-transfected with helper plasmid (pxx680) and serotype plasmid (pxx2) into HEK-293FT cells using the calcium phosphate mediated method. Forty-eight hours after transfection, 5×10^7 cells were harvested, resuspended in ice-cold PBS and extracted with chloroform. NaCl was added to the aqueous phase of the chloroform-extracted cell lysate to a final concentration of 1 M. AAV particles were then precipitated overnight by the addition of 10% (v/v) PEG-8000 powder and treated with 200 U-ml-1 benzonase, extracted again with chloroform and concentrated using a Millipore Amicon Ultra centrifugal filter (100 kDa cut-off). Viral genome-containing particles were titrated by quantitative PCR. Briefly, the prepared AAV2/9-flex-miR19b-EGFP plasmid (1 $\mu g \cdot \mu l^{-1}$) was serially diluted 10^2, 10^3, 10^4, 10^5, 10^6 fold, and used as template to generate standard curve. Primer pairs targeting CMV promoter and EGFP were used to generate CT value-$\log_{10}$(Plasmid concentration) curve. times and used as template to generate a standard curve. Primer pairs targeting the CMV promoter and EGFP were used to generate a CT value-log10(plasmid concentration) curve. The viral copy number was calculated from the plasmid concentration to viral particles:

$$\text{Viral particles } (V.G.\cdot ml^{-1}) = C_{Plasmid}\ (mg\cdot ml^{-1}) \times 10^{-3} \times 6.02 \times 10^{23}/MW_{plasmid}\ (6449 \times 648)$$

All AAV was diluted to 1×10^{13} V.G.$\cdot ml^{-1}$ and aliquoted. AAV2/9-hSyn-Cre-WPRE-pA was purchased from Shanghai Taitool Bioscience Co.Ltd..

A separate pool of HSA-sired male F1 rats was used. They underwent food training, surgery

and initial fixed-ratio training as in Experiment 5, and were equally divided into two groups to overexpress miR-19b of miR-Scramble. For AAV delivery, $AAV_{2/9}$-hSyn-Cre-WPRE-pA was mixed with $AAV_{2/9}$-flex-miR19b-EGFP or $AAV_{2/9}$-flex-Scr-EGFP to achieve equal particle numbers prior to injection. The intended stereotaxic coordinates for the nucleus accumbens were AP +2 mm; ML ±1.2 mm; DV -7 mm.

Injected animals were allowed to recover for 7 days, and subjected to heroin seeking tests under FR5 for 10 days, and PR test for one day. After behavioral testing, histological slides were examined for EGFP expression. One animals from HSA-sired F1-19b group were lost due to incorrect injection coordinate.

Experiment 14 Sperm RNA microinjection into fertilized eggs, and behavioral assessment of RNA-F1 offspring

Figures involved: Figures 3a-e

Procedure:

Total cauda epididymis sperm RNA, pooled from 3-5 samples of the same group from Experiment 2, was adjusted to a concentration of 1 $ng \cdot \mu l^{-1}$, aliquoted and stored at -80°C in a refrigerator until use.

Three weeks prior to transplantation, vasectomized male rats were prepared using 5-week-old male rats to induce pseudopregnancy in sexually mature female rats. Briefly, the rats were positioned supine, and the testes were carefully extruded from the scrotum and returned to the abdominal cavity. The abdominal cavity was accessed by incising 0.5 cm of skin on both sides of the ventral white line, 1 cm above the urethra to expose the vas deferens. The vas deferens was then separated from the surrounding connective tissue, followed by the tying of two knots at a distance of 0.5 cm from the mid-section of the vas deferens. The vas deferens was then cut from the middle of the two knots. The muscle and fat were sutured in layers, and the skin was finally sutured. The wound was treated with lincomycin (0.5%) and lidocaine (0.4%) gel (Shanghai Pharmaceuticals, Shanghai, PRC) to alleviate pain and prevent infection. Two weeks after ligation, mating training was initiated. On a three-day cycle, the ligated rats were mated with the females at 23:00 ZT, and the vaginal plugs were checked six hours later. Males that demonstrated three successful matings were considered to have completed the training and were subsequently used for mating with oocyte recipient females.

Virgin female rats aged 5 weeks were used as oocyte donors. They were super-ovulated with an

intraperitoneal injection of 20 IU PMSG (Vitro Life AB） followed by 25 IU HCG (ProSpec-Tany TechnoGene Ltd.) 48 h later.

Two days before transplantation, adopted females were mated with eight-week-old naive male rats and checked for vaginal plugs six hours later. These plugged females were then kept one in a cage until they gave birth. This was to ensure that pups from surrogate females are between PND 0 and 5 when caesarean sections are performed on transplanted females.

The day before transplantation, two separate batches of mating was carried out, to produce oocyte donor rats, surrogate females. For the production of oocyte donors, the super-ovulated females were mated with eight-week-old naïve male rats and checked 6 h later. Females with vaginal plugs were euthanized with isofluorane overdose, the abdominal cavity was opened, and the oviducts were separated and placed in pre-warmed M2 culture solution (Merck KGaA) at 37°C

Subsequently, operating under a dissecting microscope, a 1 ml syringe was lanced through the fimbriae of oviduct and a small amount of M2 culture fluid was pushed in to allow the fertilized eggs to naturally flow out of the tube. These eggs were transferred to clean M2 culture medium with a mouth pipette, then hyaluronidase was added to wash off the granular cells. The eggs were sequentially transferred to clean M2 culture medium drops until the granulocytes were removed, and then transferred to G-IVF culture medium (Vitro Life AB), and placed in 37°C 5% CO2 cell culture incubator.

RNA was injected in the cytoplasm of fertilized zygotes using home-made injector (Parameters for needle pulling with P-97, Heat = 549°C, Pull = 200, Velocity = 80, Time = 175). Injected

zygotes were then used for transplantation.

The day after mating with vasectomized males, surrogate female rats with copulatory plugs were used as recipients. During zygote transfer, they were maintained under isoflurane anesthesia (2.5%) with an incision on the back to expose the ovary. Approximately 20 zygotes were then transferred from the oviductal ampulla into one side of the oviduct using a mouse pipette. An air bubble was blown into the oviduct to prevent the transplanted eggs from falling out. The muscle and fat were sutured in layers and the skin was sutured at the end. The wound was treated with lincomycin (0.5%) and lidocaine (0.4%) gel (Shanghai Pharmaceuticals) to relieve pain and prevent infection. These surrogate female rats were kept in a cage until the end of pregnancy.

If surrogate female rats were unable to give birth normally 20 days after mating, pups were delivered by caesarean section. On a thermal preservation mat, surrogate female rats were euthanized with isoflurane overdose, and the uterus was then exposed and removed. The pups were removed, the placenta was removed after the umbilical cord was closed with a hemostatic electrocoagulation pen, and the pups were cleaned with sterile gauze until they were bright red all over and could breathe freely. They were maintained in the bedding of surrogate females for 30 min, and returned to the cage of surrogate females, and weaned at 21 days.

RNA-F1 rats of the same sex were maintained 3-4 per cage until 8 weeks of age. They were then randomly assigned for sucrose self-administration or heroin self-administration tests as in Experiments 5 and 7.

Experiment 15 Effect of miR-19b normalization of sperm RNA on heroin-seeking behavior of RNA-F1 offsrping

Figures involved: Figure S6, Figure 5a-b

Procedure:

For co-injection of sperm RNA with synthetic miR-19b in fertilized eggs, absolute quantification of miR-19b in each sperm RNA sample was first performed. First, a standard curve was generated. CDNA was synthesized with 1 nM of synthetic miR-19b RNA mimics, serially diluted in 10-fold gradient for 10, 10^2, 10^3, 10^4, 10^5-fold, and used as template to generate standard curves. RNA samples from Experiment 3 were used for absolute miR-19b quantification by reverse transcription of 50 ng sperm RNA. The the difference between HSA and SSA sperm was determined, and supplemented with synthetic agomir-19b (Genepharma, China). The microinjection, transplantation, and subsequent behavioral assay of RNA-F1 was done as in Experiment 14.

Experiment 16 Validation of AAV-based miR-19b knockdown, and behavioral assessment of NAc miR-19b knockdown on heroin self-administration in naïve male SD rats

Figures involved: Figure S8, Figure 5f

Procedure:

A Cre-dependent miRNA suppression plasmid expressing the decoy TuD (tough decoy) RNA[30] was amplified by PCR (primers are provided in Supplementary Table 2) and constructed in EcoRI/XhoI linearised pAKD-CMV-bGlobin-Flex-EGFP-MIR30shRNA vector using the MultiS One Step Cloning Kit (Vazyme Biotech Co., Ltd., China) according to the manufacturer's instructions. The subcloned plasmid was transduced into DH5a competent cells and validated by Sanger sequencing.

The verification of miR-19b knockdown by TuD-19b was performed by luciferase assay. For the luciferase assay, synthetic oligonucleotides encoding perfect miRNA target sites against miR-19b or scramble sequence (miR-19b, AGTTTTGCAGATTTGCAGTTCAG, scramble, GAGCAATTATTGTGTTGCCGTT) were inserted into XhoI/NotI-digested psiCHECK-2 (Promega Co. Ltd., USA), targeting the 3'UTR of the synthetic Renilla luciferase gene. The plasmids were then prepared using the ZymoPURE™ II Plasmid Maxiprep Kit (Zymo Research) and used for transfection.

HEK 293T cells were seeded at a density of 1.2×10^5 cells per well in 24-well plates in DMEM containing 10% FBS the day before transfection. Cells were transfected using calcium phosphate/DNA co-precipitation method with a combination of 100 ng of psiCHECK-2 containing miR-19b or control site, 500 ng of TuD RNA expression plasmid. Dual-Luciferase® Reporter Assay System (Promega) was used to assess the efficiency of firefly and

Renilla expression 48 hours after transfection using GLOMAX (Promega).

AAV production and tittering was done in accordance to Experiment 13. Naïve male SD rats were used. They underwent food training, surgery and initial fixed-ratio training as in Experiment 5, and were equally divided into two groups to overexpress TuD-miR-19b or TuD-Scramble. For AAV delivery, $AAV_{2/9}$-hSyn-Cre-WPRE-pA was mixed with $AAV_{2/9}$-flex-TuD-miR-19b-EGFP or $AAV_{2/9}$-flex-TuD-Scr-EGFP to achieve equal particle numbers prior to injection. The intended stereotaxic coordinates for the nucleus accumbens were AP +2 mm; ML ±1.2 mm; DV -7 mm. Injected animals were allowed to recover for 7 days, and subjected to heroin seeking tests under FR5 for 10 days, and PR test for one day. After behavioral testing, histological slides were examined for EGFP expression with 1 animal excluded.

Experiment 17 Overexpression of miR-19b in NAc of naïve male SD rats to assess miR-19b level of various tissues

Figures involved: Figures S9-10, Figure 11a,c-e

Procedure:

For AAV delivery, $AAV_{2/9}$-hSyn-Cre-WPRE-pA was mixed with $AAV_{2/9}$-flex-TuD-miR-19b-EGFP or $AAV_{2/9}$-flex-TuD-Scr-EGFP to achieve equal particle numbers of 5×10^{12} v.g./ml prior to injection. Naïve male Sprague-Dawley (SD) rats, aged 8 weeks, were used for the experiments. Animals were anesthetized with isoflurane (2.5%) and secured in a stereotaxic apparatus. The incisor bar was adjusted to ensure that the heights of lambda and bregma were equal (±0.1 mm). A sharp scalpel was used to make a midline incision in the scalp, exposing the anterior and posterior fontanelles and the injection area. Sterilized cotton balls were

employed to gently remove and stanch any bleeding from the cranial surface tissue. Using a micro-drill, a small hole was carefully drilled at the target coordinates for the nucleus accumbens (NAc): AP +2 mm; ML ±1.2 mm; DV -7 mm. Glass pipettes connected to a Nanoject III microinjector (Drummond Scientific Co., USA) were used for virus delivery. The pipette was slowly lowered to the predetermined depth (DV -7 mm). A total of 1.2 μL of AAV viral solution was infused at a rate of 0.24 μL/min over a period of 5 minutes. After the infusion, the glass pipette was left in place for an additional 5 minutes to allow for sufficient diffusion of the virus into the surrounding brain tissue. The scalp incision was closed using sterile sutures to minimize infection risk. For anti-infection and pain management, lincomycin (0.5%) and lidocaine (0.4%) gel (Shanghai Pharmaceuticals, China) was applied to the incision area. Animals were then placed in a warm, quiet recovery area until they fully regained consciousness. Post-operative monitoring included daily anti-infection and pain management for the first three days, regular checks on the animals' behavior, wound healing, and overall health status.

At indicated time points (1, 2, 4 weeks post), rats were euthanized with an overdose of isoflurane, for sampling of blood, brain, liver, lymph nodes, spleen, testis, caput and cauda epididymis. First, the thoracic cavity was opened in order to expose the heart. Blood was collected by cardiac puncture using sodium citrate collection tubes. The collected blood was immediately centrifuged at 800g for 15 minutes, then 2,500g for 20 minutes and 10,000g for 30 minutes at 4°C to obtain platelet-free plasma, which was aliquoted and stored at -80°C until use. Then the right auricle of the rat was clipped, and 50 mL of ice-cold saline was perfused into the left ventricle with an 18-gauge syringe needle. Subsequently, 3 mm × 3 mm × 3 mm cubicle of liver, as well as spleen were removed. Then

internal iliac lymph nodes were collected. These tissues were Dounce homogenized in ice-cold PBS and divided into two parts. One was used for RNA isolation, which was mixed with 10 times of Trizol reagent, homogenized, and stored at -20°C until RNA isolation. The other was frozen at -80°C for protein level assay and viral DNA extraction.

The brain, bilateral epididymis and vas deferens were removed and dissected on ice for sampling, as described in Experiment 2. The brain was digested with Hibernate-A solution with Collagenase III (75 U/mL) with gentle titration every 3 minutes for 15 min. For isolation of testicular spermatozoa, each testis was digested with 5 mL elutriation buffer (100 mM NaCl, 45 mM KCl, 6 mM Na_2HPO_4, 0.6 mM KH_2PO_4, 0.23% sodium DL-lactate, 0.1% glucose, 0.1% BSA, 0.011% sodium pyruvate, 1.2 mM $MgSO_4$ and 1.2 mM $CaCl_2$) with 25 $\mu g \cdot ml^{-1}$ Liberase™ (Roche Diagnostics GmbH, Germany) for 30 min at 37°C with gentle agitation every 5 min, followed by titration. Caput and cauda epididymal spermatozoa were collected by making incisions at both ends of the tissue and flushing the seminiferous tubules with pre-warmed saline using a 26-gauge needle. The flushed cauda epididymis was further minced and incubated at 37°C for 10 minutes, and the top 2 mL of supernatant was transferred to a new conical tube.

The above suspension was filtered twice through a 40-µm cell strainer on ice, and divided as above. Half of the suspension was frozen at -80°C for protein level assay and viral DNA extraction. The other half was then centrifuged at 800 g at 4°C for 10 min. The pellets of the NAc and mPFC were then homogenized in Trizol, and stored at -20°C. The supernatant was centrifuged at 3,000 g for 20 min, filtered with a 0.22 µm filter, and then mixed with 1/3 volume of VEX Exosome Isolation Reagent from cell culture media (Vazyme), and supplied with 0.5% RNAse inhibitor (Takara), kept at 4°C overnight.

Blood exosome was collected by mixing with 1/5 volume of VEX Exosome Isolation Reagent (from serum), and kept at 4°C for 30 min. Exosome were collected by centrifugation at 10,000 g for 30 min at 4°C, and resuspended in Trizol reagent until use.

RNA extraction and reverse transcription was done as in Experiment 3 and Experiment 4. Viral DNA extraction was done by using the FastPure Viral DNA/RNA Mini Kit (Vazyme). Protein level of each sample was assayed using Pierce BCA Protein Assay Kit (Thermofischer).

For tittering of viral copy number of each tissue, the prepared plasmid (1 $\mu g \cdot \mu l^{-1}$) was serially diluted 10^2, 10^3, 10^4, 10^5, 10^6-fold, and used as template to generate standard curves for each viral DNA components. Primer pairs targeting viral components (CMV from AAV2/9-flex-miR19b-EGFP, hSyn and Cre from AAV2/9-hSyn-Cre-WPRE-pA). were used to generate CT value-$\log_{10}$(Plasmid concentration) curve. The standard curve was generated by plotting the $\log_{10}$ concentration versus Ct. The curve was then used for quantification of viral genome-containing particle of prepared AAV or from tissue samples. Blood AAV titers were expressed as V.G./ml, and viral loads in tissue were presented as V.G./mg protein.

Experiment 18 Intra cerebral ventricle GW4869 infusion to assess miR-19b level of various tissues

Figures involved: Figure S11b

Procedure:

Eight-week-old male rats were subjected to cannula implantation. Briefly, rats were anaesthetized with isoflurane (2.5%) and placed in a stereotaxic instrument (RWD Life Science). The skin on the top of the head was cut with a sharp scalpel to expose the skull, and the incisor bar was adjusted until the lambda and bregma heights were equal. A cannula

(PlasticsOne, 22GA, 5 mm) was implanted in the skull with the following coordinates: AP -0.8 mm; ML 1.5 mm; DV -4 mm and fixed to the skull with dental cements. The incision was then treated with lincomycin (0.5%) and lidocaine (0.4%) gel (Shanghai Pharmaceuticals, Shanghai, PRC) for pain relief and anti-infective treatment for seven days.

For GW4869 administration, intracerebroventricular injection of GW4869 (3 μL of 50 nM in 10% DMSO, MCE; administered over 6 min and allowed an additional 4 min for sufficient diffusion) or vehicle was performed for four consecutive days, with an insertion needle reaching DV -5 mm. On the fifth day, the rats were sacrificed and their tissues were collected for RNA extraction and qPCR as performed in previous experiments.

1 Green, M. R. & Sambrook, J. Isolation of DNA Fragments from Polyacrylamide Gels by the Crush and Soak Method. *Cold Spring Harbor Protocols* **2019**, pdb.prot100479 (2019). https://doi.org/10.1101/pdb.prot100479

2 Gaspar, J. M. NGmerge: merging paired-end reads via novel empirically-derived models of sequencing errors. *BMC Bioinformatics* **19**, 536 (2018). https://doi.org/10.1186/s12859-018-2579-2

3 Shi, J., Ko, E. A., Sanders, K. M., Chen, Q. & Zhou, T. SPORTS1.0: A Tool for Annotating and Profiling Non-coding RNAs Optimized for rRNA- and tRNA-derived Small RNAs. *Genomics Proteomics Bioinformatics* **16**, 144-151 (2018). https://doi.org/10.1016/j.gpb.2018.04.004

4 Griffiths-Jones, S., Grocock, R. J., van Dongen, S., Bateman, A. & Enright, A. J. miRBase: microRNA sequences, targets and gene nomenclature. *Nucleic Acids Res* **34**, D140-144 (2006). https://doi.org/10.1093/nar/gkj112

5 Langmead, B., Trapnell, C., Pop, M. & Salzberg, S. L. Ultrafast and memory-efficient alignment of short DNA sequences to the human genome. *Genome Biol* **10**, R25 (2009). https://doi.org/10.1186/gb-2009-10-3-r25

6 Grosjean, H., de Crécy-Lagard, V. & Marck, C. Deciphering synonymous codons in the three domains of life: co-evolution with specific tRNA modification enzymes. *FEBS Lett* **584**, 252-264 (2010). https://doi.org/10.1016/j.febslet.2009.11.052

7 Love, M. I., Huber, W. & Anders, S. Moderated estimation of fold change and dispersion for RNA-seq data with DESeq2. *Genome Biol* **15**, 550 (2014). https://doi.org/10.1186/s13059-014-0550-8

8 Cahill, K. M., Huo, Z., Tseng, G. C., Logan, R. W. & Seney, M. L. Improved identification of concordant and discordant gene expression signatures using an updated rank-rank hypergeometric overlap approach. *Sci Rep* **8**, 9588 (2018). https://doi.org/10.1038/s41598-018-27903-2
9 Kern, F. *et al.* miEAA 2.0: integrating multi-species microRNA enrichment analysis and workflow management systems. *Nucleic Acids Res* **48**, W521-w528 (2020). https://doi.org/10.1093/nar/gkaa309
10 Wang, T., Gu, J. & Li, Y. Inferring the perturbed microRNA regulatory networks from gene expression data using a network propagation based method. *BMC Bioinformatics* **15**, 255 (2014). https://doi.org/10.1186/1471-2105-15-255
11 Li, C. Y., Mao, X. & Wei, L. Genes and (common) pathways underlying drug addiction. *PLoS Comput Biol* **4**, e2 (2008). https://doi.org/10.1371/journal.pcbi.0040002
12 Yan, B. *et al.* MiR-218 targets MeCP2 and inhibits heroin seeking behavior. *Sci Rep* **7**, 40413 (2017). https://doi.org/10.1038/srep40413
13 Niu, Y. *et al.* An improved method for detecting circulating microRNAs with S-Poly(T) Plus real-time PCR. *Sci Rep* **5**, 15100 (2015). https://doi.org/10.1038/srep15100
14 Bolger, A. M., Lohse, M. & Usadel, B. Trimmomatic: a flexible trimmer for Illumina sequence data. *Bioinformatics* **30**, 2114-2120 (2014). https://doi.org/10.1093/bioinformatics/btu170
15 Kim, D., Paggi, J. M., Park, C., Bennett, C. & Salzberg, S. L. Graph-based genome alignment and genotyping with HISAT2 and HISAT-genotype. *Nat Biotechnol* **37**, 907-915 (2019). https://doi.org/10.1038/s41587-019-0201-4
16 Liao, Y., Smyth, G. K. & Shi, W. featureCounts: an efficient general purpose program for assigning sequence reads to genomic features. *Bioinformatics* **30**, 923-930 (2014). https://doi.org/10.1093/bioinformatics/btt656
17 Yu, G., Wang, L. G., Han, Y. & He, Q. Y. clusterProfiler: an R package for comparing biological themes among gene clusters. *OMICS* **16**, 284-287 (2012). https://doi.org/10.1089/omi.2011.0118
18 McGeary, S. E. *et al.* The biochemical basis of microRNA targeting efficacy. *Science* **366** (2019). https://doi.org/10.1126/science.aav1741
19 Bindea, G. *et al.* ClueGO: a Cytoscape plug-in to decipher functionally grouped gene ontology and pathway annotation networks. *Bioinformatics* **25**, 1091-1093 (2009). https://doi.org/10.1093/bioinformatics/btp101
20 Chang, K., Marran, K., Valentine, A. & Hannon, G. J. Creating an miR30-based shRNA vector. *Cold Spring Harb Protoc* **2013**, 631-635 (2013). https://doi.org/10.1101/pdb.prot075853
21 Haraguchi, T., Ozaki, Y. & Iba, H. Vectors expressing efficient RNA decoys achieve the long-term suppression of specific microRNA activity in mammalian cells. *Nucleic Acids Res* **37**, e43 (2009). https://doi.org/10.1093/nar/gkp040

References

1 O'Brien, E. A., Ensbey, K. S., Day, B. W., Baldock, P. A. & Barry, G. Direct evidence for transport of RNA from the mouse brain to the germline and offspring. *BMC Biol* **18**, 45 (2020). https://doi.org/10.1186/s12915-020-00780-w

2 Men, Y. *et al.* Exosome reporter mice reveal the involvement of exosomes in mediating neuron to astroglia communication in the CNS. *Nat Commun* **10**, 4136 (2019). https://doi.org/10.1038/s41467-019-11534-w

3 Qing, L., Chen, H., Tang, J. & Jia, X. Exosomes and Their MicroRNA Cargo: New Players in Peripheral Nerve Regeneration. *Neurorehabil Neural Repair* **32**, 765-776 (2018). https://doi.org/10.1177/1545968318798955

4 Reilly, J. N. *et al.* Characterisation of mouse epididymosomes reveals a complex profile of microRNAs and a potential mechanism for modification of the sperm epigenome. *Sci Rep* **6**, 31794 (2016). https://doi.org/10.1038/srep31794

5 Dai, Y. *et al.* The distribution of nerves supplying the testis, epididymis and accessory sex glands of Suncus murinus. *Anat Sci Int* **94**, 128-135 (2019). https://doi.org/10.1007/s12565-018-0459-5

6 Ricker, D. D., Chamness, S. L., Hinton, B. T. & Chang, T. S. Changes in luminal fluid protein composition in the rat cauda epididymidis following partial sympathetic denervation. *J Androl* **17**, 117-126 (1996).

7 Gerendai, I. Modulation of testicular functions by testicular opioid peptides. *Journal of physiology and pharmacology : an official journal of the Polish Physiological Society* **42**, 427-437 (1991).

8 Schiapparelli, L. M. *et al.* Proteomic screen reveals diverse protein transport between connected neurons in the visual system. *Cell Rep* **38**, 110287 (2022). https://doi.org/10.1016/j.celrep.2021.110287

9 D'Arrigo, G. *et al.* Astrocytes-derived extracellular vesicles in motion at the neuron surface: Involvement of the prion protein. *Journal of extracellular vesicles* **10**, e12114 (2021). https://doi.org/10.1002/jev2.12114

10 Green, M. R. & Sambrook, J. Isolation of DNA Fragments from Polyacrylamide Gels by the Crush and Soak Method. *Cold Spring Harbor Protocols* **2019**, pdb.prot100479 (2019). https://doi.org/10.1101/pdb.prot100479

11 Gaspar, J. M. NGmerge: merging paired-end reads via novel empirically-derived models of sequencing errors. *BMC Bioinformatics* **19**, 536 (2018). https://doi.org/10.1186/s12859-018-2579-2

12 Shi, J., Ko, E. A., Sanders, K. M., Chen, Q. & Zhou, T. SPORTS1.0: A Tool for Annotating and Profiling Non-coding RNAs Optimized for rRNA- and tRNA-derived Small RNAs. *Genomics Proteomics Bioinformatics* **16**, 144-151 (2018). https://doi.org/10.1016/j.gpb.2018.04.004

13 Griffiths-Jones, S., Grocock, R. J., van Dongen, S., Bateman, A. & Enright, A. J. miRBase: microRNA sequences, targets and gene nomenclature. *Nucleic Acids Res* **34**,

D140-144 (2006). https://doi.org/10.1093/nar/gkj112

14 Langmead, B., Trapnell, C., Pop, M. & Salzberg, S. L. Ultrafast and memory-efficient alignment of short DNA sequences to the human genome. *Genome Biol* **10**, R25 (2009). https://doi.org/10.1186/gb-2009-10-3-r25

15 Grosjean, H., de Crécy-Lagard, V. & Marck, C. Deciphering synonymous codons in the three domains of life: co-evolution with specific tRNA modification enzymes. *FEBS Lett* **584**, 252-264 (2010). https://doi.org/10.1016/j.febslet.2009.11.052

16 Love, M. I., Huber, W. & Anders, S. Moderated estimation of fold change and dispersion for RNA-seq data with DESeq2. *Genome Biol* **15**, 550 (2014). https://doi.org/10.1186/s13059-014-0550-8

17 Cahill, K. M., Huo, Z., Tseng, G. C., Logan, R. W. & Seney, M. L. Improved identification of concordant and discordant gene expression signatures using an updated rank-rank hypergeometric overlap approach. *Sci Rep* **8**, 9588 (2018). https://doi.org/10.1038/s41598-018-27903-2

18 Kern, F. *et al.* miEAA 2.0: integrating multi-species microRNA enrichment analysis and workflow management systems. *Nucleic Acids Res* **48**, W521-w528 (2020). https://doi.org/10.1093/nar/gkaa309

19 Wang, T., Gu, J. & Li, Y. Inferring the perturbed microRNA regulatory networks from gene expression data using a network propagation based method. *BMC Bioinformatics* **15**, 255 (2014). https://doi.org/10.1186/1471-2105-15-255

20 Li, C. Y., Mao, X. & Wei, L. Genes and (common) pathways underlying drug addiction. *PLoS Comput Biol* **4**, e2 (2008). https://doi.org/10.1371/journal.pcbi.0040002

21 Yan, B. *et al.* MiR-218 targets MeCP2 and inhibits heroin seeking behavior. *Sci Rep* **7**, 40413 (2017). https://doi.org/10.1038/srep40413

22 Niu, Y. *et al.* An improved method for detecting circulating microRNAs with S-Poly(T) Plus real-time PCR. *Sci Rep* **5**, 15100 (2015). https://doi.org/10.1038/srep15100

23 Bolger, A. M., Lohse, M. & Usadel, B. Trimmomatic: a flexible trimmer for Illumina sequence data. *Bioinformatics* **30**, 2114-2120 (2014). https://doi.org/10.1093/bioinformatics/btu170

24 Kim, D., Paggi, J. M., Park, C., Bennett, C. & Salzberg, S. L. Graph-based genome alignment and genotyping with HISAT2 and HISAT-genotype. *Nat Biotechnol* **37**, 907-915 (2019). https://doi.org/10.1038/s41587-019-0201-4

25 Liao, Y., Smyth, G. K. & Shi, W. featureCounts: an efficient general purpose program for assigning sequence reads to genomic features. *Bioinformatics* **30**, 923-930 (2014). https://doi.org/10.1093/bioinformatics/btt656

26 Yu, G., Wang, L. G., Han, Y. & He, Q. Y. clusterProfiler: an R package for comparing biological themes among gene clusters. *OMICS* **16**, 284-287 (2012). https://doi.org/10.1089/omi.2011.0118

27 McGeary, S. E. *et al.* The biochemical basis of microRNA targeting efficacy. *Science* **366** (2019). https://doi.org/10.1126/science.aav1741

28 Bindea, G. *et al.* ClueGO: a Cytoscape plug-in to decipher functionally grouped gene ontology and pathway annotation networks. *Bioinformatics* **25**, 1091-1093 (2009).

https://doi.org/10.1093/bioinformatics/btp101

29 Chang, K., Marran, K., Valentine, A. & Hannon, G. J. Creating an miR30-based shRNA vector. *Cold Spring Harb Protoc* **2013**, 631-635 (2013). https://doi.org/10.1101/pdb.prot075853

30 Haraguchi, T., Ozaki, Y. & Iba, H. Vectors expressing efficient RNA decoys achieve the long-term suppression of specific microRNA activity in mammalian cells. *Nucleic Acids Res* **37**, e43 (2009). https://doi.org/10.1093/nar/gkp040

Supplementary figures

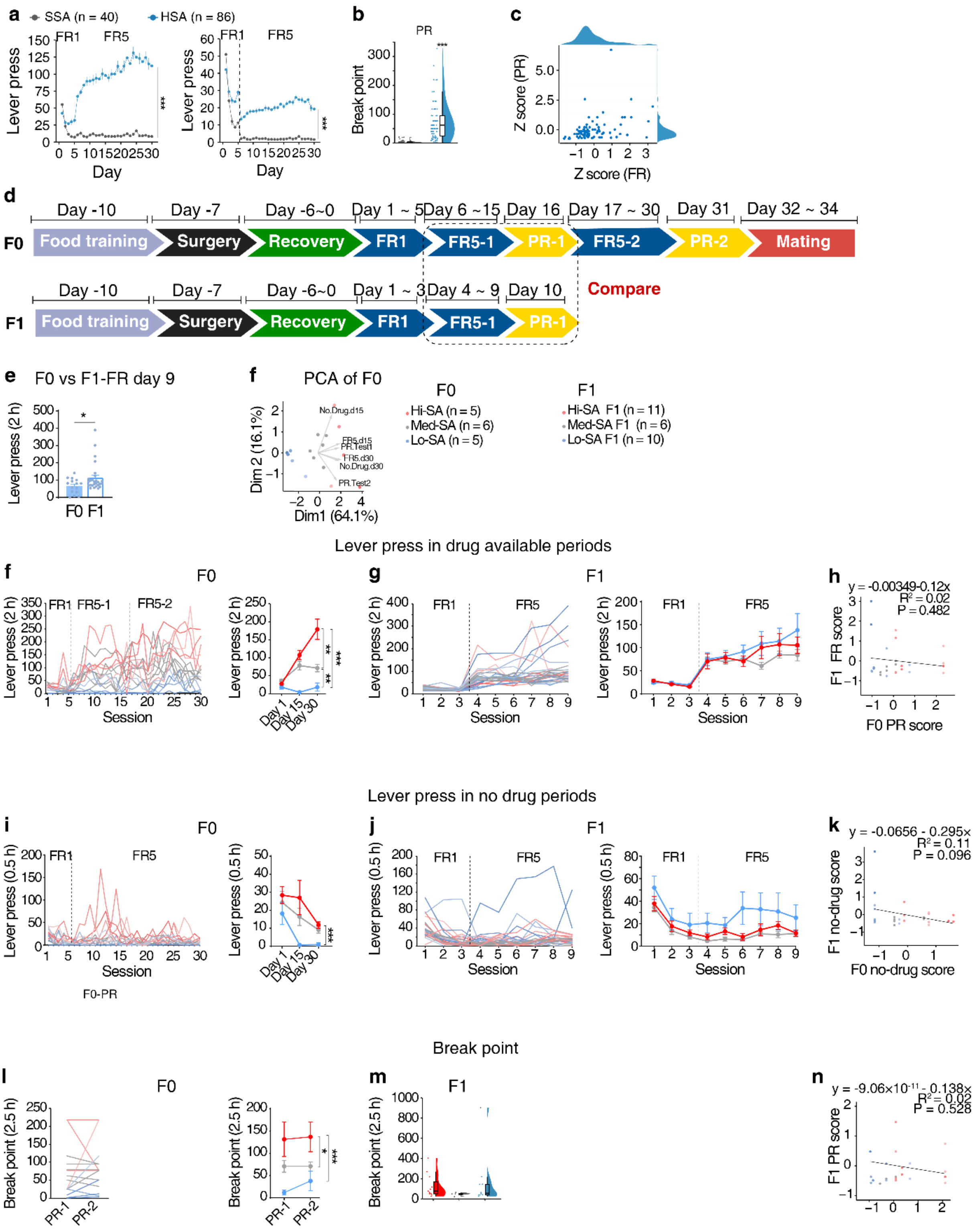

Figure S1. Performance of rat heroin self-administration of HSA and SSA groups and potential effects of paternal individual differences on offspring. **(a-c)** Lever pressed in FR

sessions (**a**), infusions in FR sessions (**b**), and breakpoint in PR session (**c**) in heroin SA tests of naïve male rats used in the study. (**d-n**) F0-F1 correlation analysis. (**d**) Schematics for F0 and F1 heroin self-administration tests. Sixteen naïve male SD rats were subjected to heroin self-administration training under a consecutive 5-day FR1, 10-day FR5, followed by 1-day PR test (PR-1), and then 15-day FR5 and1-day PR reinforcement schedule (PR-2). Then they were co-housed with naïve female rats to generate F1. F0-F1 correlation analysis were carried out using their performances in FR and PR schedules. (**e**) Comparison of F0-F1 performance on lever pressed on day 9. **(f)** PCA analysis of the 16 rats based on performance of lever presses on FR5-Day15, FR5-Day31, PR-Test 1, PR-Test 2, no-drug-Day15, and no-drug-Day30. **(g)** Individual performance (left) and averaged SA performance of Hi-SA, Med-SA, and Lo-SA on fixed-ratio session 1,15, and 31. **(h)** Individual performance (left) and averaged lever pressed on fixed-ratio sessions of F1 offspring. (**i**) F0-F1 correlation of lever pressed during FR sessions. (**j**) Individual performance (left) and averaged lever pressed of Hi-SA, Med-SA, and Lo-SA on no-drug session 1,15, and 31. (**k**) Individual performance (left) and averaged SA performance of F1 offspring sired by Hi-SA, Med-SA, and Lo-SA on no-drug sessions. (**i**) F0-F1 correlation of lever pressed during no-drug sessions. **(l)** Individual performance (left) and averaged lever pressed of Hi-SA, Med-SA, and Lo-SA on progressive-ratio tests. (**m**) Individual performance (left) and averaged lever pressed of F1 offspring sired by Hi-SA, Med-SA, and Lo-SA on progressive-ratio tests. **(n)** F0-F1 correlation of PR scores. For FR tests, results are presented as mean ± s.e.m in the line plot. For PR tests, polygon represents density estimates of data, the box represents the 25th and 75th percentiles, the

whiskers show the minimum and maximum of the data, and the line inside the box denotes the median. *$P < 0.05$, **$P < 0.01$, ***$P < 0.001$.

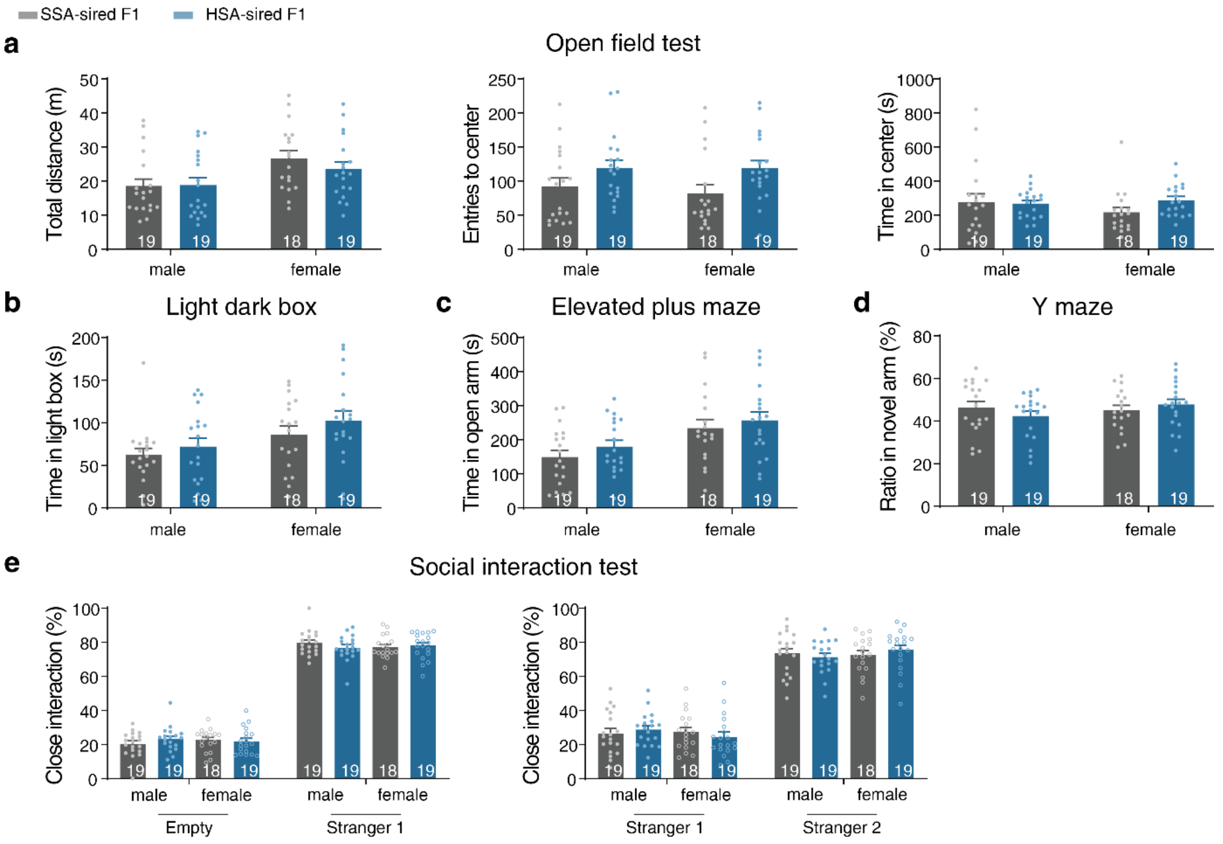

Figure S2. Comparable performance of HSA-sired F1 and SSA-sired F1 rats in open field, light-dark box, elevated plus maze, Y maze, and social interaction tests.

(**a**) Open field test of F1 offspring. Distance travelled, entries to the center area, and time spent in the center area were recorded. (**b**) Time spent in the light box of light-dark box shuttle test of F1 offspring. (**c**) Time spent in the open arm of plus maze test of F1 offspring. (d) Ratio of time spent in the open arm of Y maze of F1 offspring. (e) Social interaction test of F1 offspring. Ratio of time spent in close action of sociability test, and social recognition test were recorded. Results are shown as mean ± s.e.m.

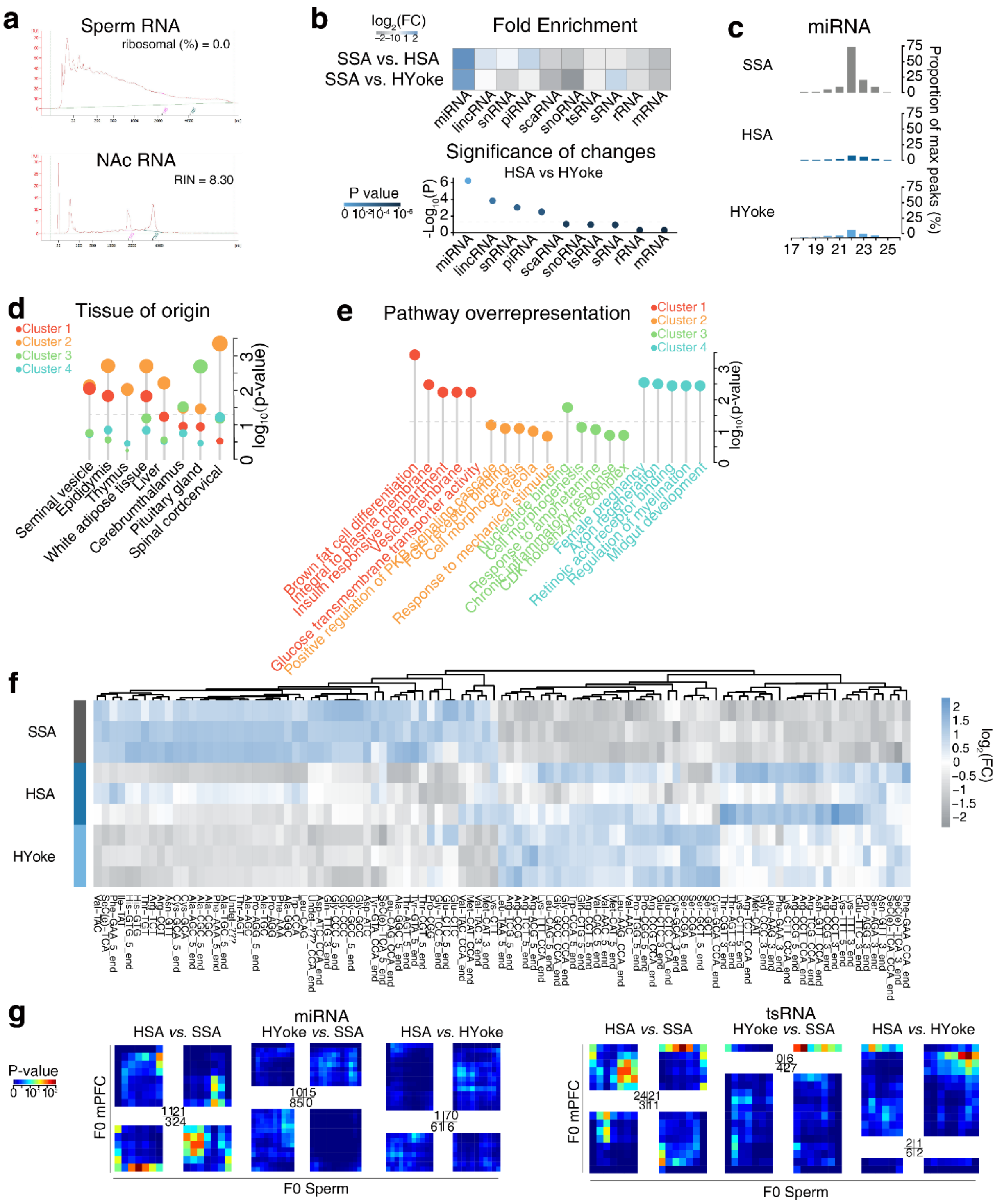

Figure S3. Expression profiling of sperm non-coding RNAs. (**a**) RNA quality control of sperm and NAc RNA. (**b**) Fold enrichment (upper) and significance (lower) of each type of small non-coding RNAs in sperm. Comparisons are made between HSA vs. SSA (upper lane) and HYoke vs. SSA (lower lane). (**c**) Enrichment of miRNA in sperm of SSA, HSA, and HYoke

groups. The values are normalized by the fraction of total reads mapped to SSA. (d-e) Tissue of origin (**d**) and top 5 pathways (**e**) of each miRNA cluster, as predicted by miEAA. (**f**) Heatmap and hierarchical clustering of differentially expressed tsRNAs between SSA, HSA and HYoke of F0 sperm. (**g**) Rank Rank Hypergeometric Overlap (RRHO) analysis of differentially-expressed miRNAs and tsRNAs in the medial prefrontal cortex (mPFC) vs. sperm of F0 generation among three groups The upper-right and lower-left region of each plot represents co- up/down regulation, while the upper-left and lower-right regions shows inconsistent changes. Color denotes significance.

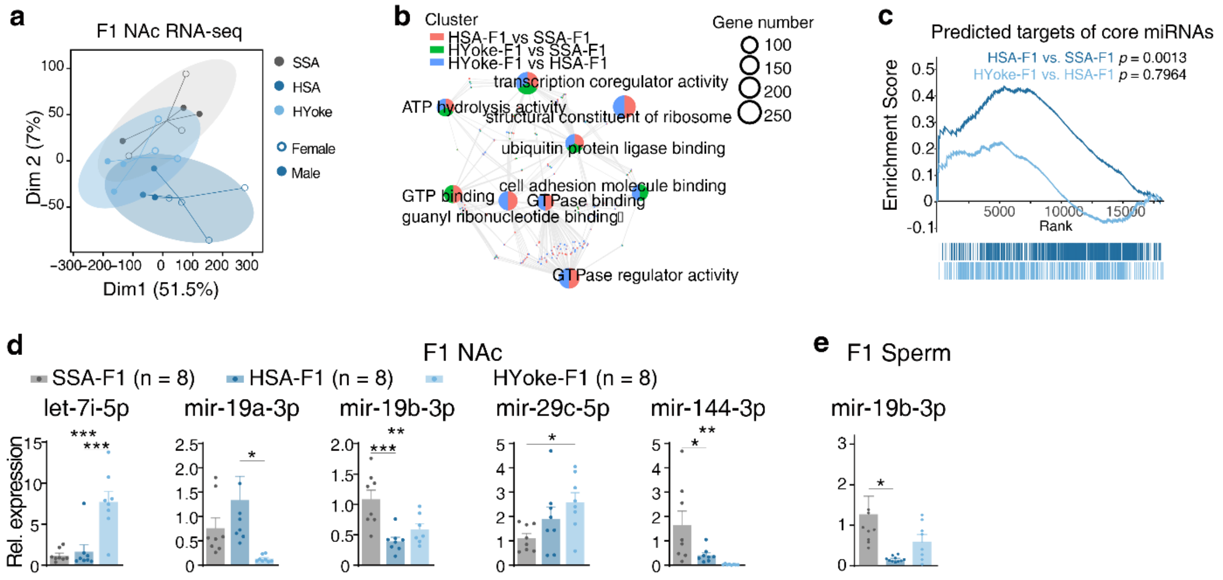

Figure S4. Transcriptomic profiling of F1 NAc. (**a**) PCA plot of the nucleus accumbens from F1 of SSA, HSA, and HYoke groups. (**b**) Pathway overrepresentation of differentially expressed genes in HSA F1 vs. SSA F1, HYoke F1 vs. SSA F1, HYoke F1 vs. HSA F1. (**c**) Gene set enrichment analysis of predicted targets of core miRNAs revealed in Fig. 4f. (**d**) Relative expression of candidate miRNAs, let-7a-5p, miR-181d-5p, miR-19b-3p, and miR-144-3p in F1 NAc by qRT-PCR. (**e**) Sperm miR-19b expression of F1 male offspring. SSA F1, female n = 6, male n = 6; HSA F1, female n = 5, male n = 7, HYoke F1, female n = 4, male n = 4. Values are expressed as mean ± s.e.m. *$P < 0.05$, **$P < 0.01$, ***$P < 0.001$.

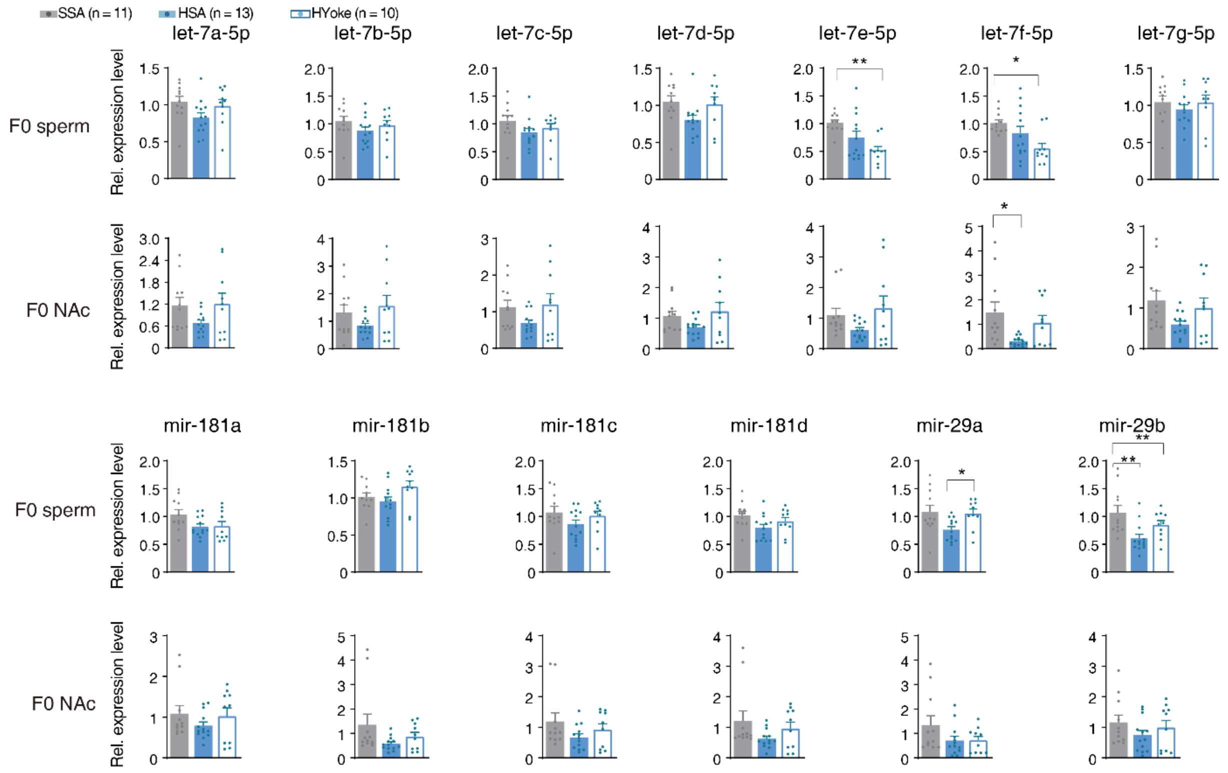

Figure S5. Validation of miRNA expression in F0 sperm and NAc by qPCR. Relative expression of candidate miRNAs, let-7b-5p, let-7c-5p, let-7d-5p, let-7e-5p, let-7f-5p, let-7g-5p, let-7i-5p, miR-19a, miR-181a, miR-181b, miR-181c, miR-29a, miR-29b, and miR-29c in F0 NAc and sperm by qRT-PCR. Values are expressed as mean ± s.e.m. One-way ANOVA followed by Bonferroni's *post hoc* test. Values are expressed as mean ± s.e.m.

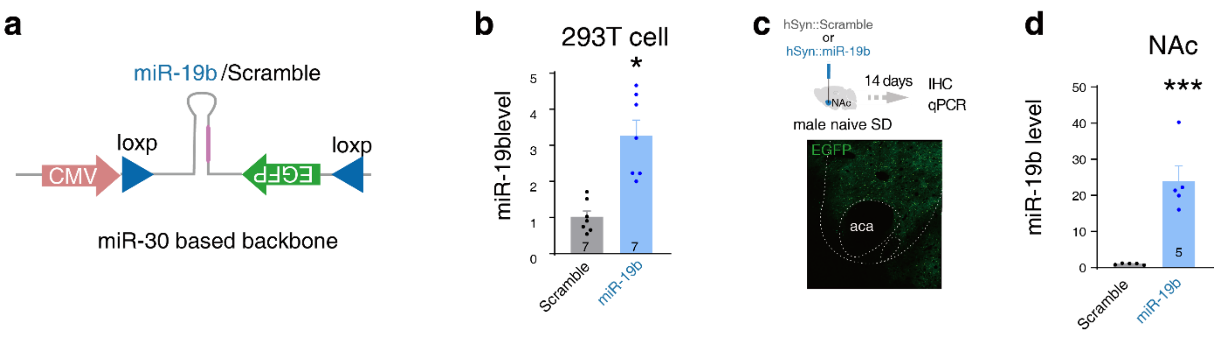

Figure S6. Validation of miR-19b over-expression vector. (**a**) Design of miR-30 backbone-based miR-19b over-expression plasmid. (**b**) Validation of miR-19b expression in 293T cell by co-transfection with CAG-Cre. (**c**) Representative image of AAV expression in the NAc revealed by EGFP expression after 2 weeks of injection. (**d**) Quantitative PCR analysis of miR-19b level after co-injection of AAV-flox-miR-19b with AAV-hSyn-Cre. Values are expressed as mean ± s.e.m. *$P < 0.05$, ***$P < 0.001$.

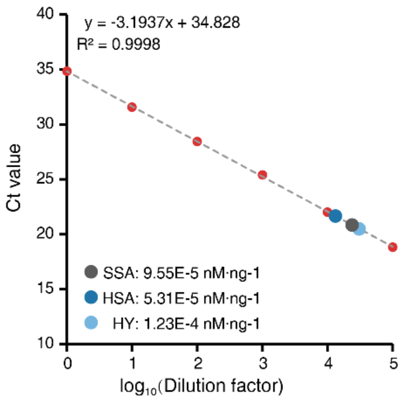

Figure S7. Absolute quantification of miR-19b of F0 sperm by qPCR. CDNA was synthesized with 1 nM of synthetic miR-19b RNA mimics, and serially diluted in 10-fold gradient to produce the standard curve. The copy number of each miRNA in sperm was calculated as nM per ng sperm RNA.

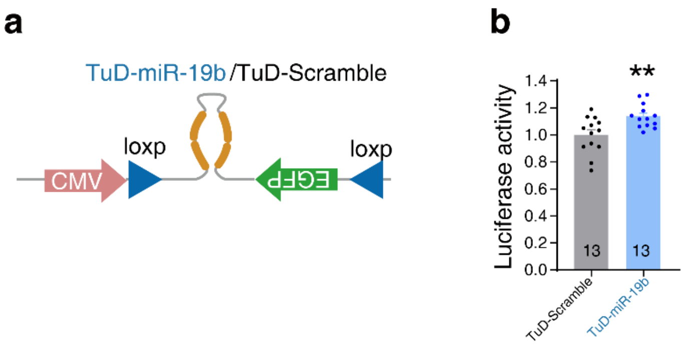

Figure S8. Validation of miR-19b down-regulation with TuD-miR-19b. (**a**) Design of miR-30 backbone-based miR-19b down-expression plasmid, TuD-19b. (**b**) Relative luciferase activity with the miR-19b target site in 3′ UTR was determined in 293 T cells co-transfected with scramble or miR-19b. Values are expressed as mean ± s.e.m. $^{**}P < 0.01$.

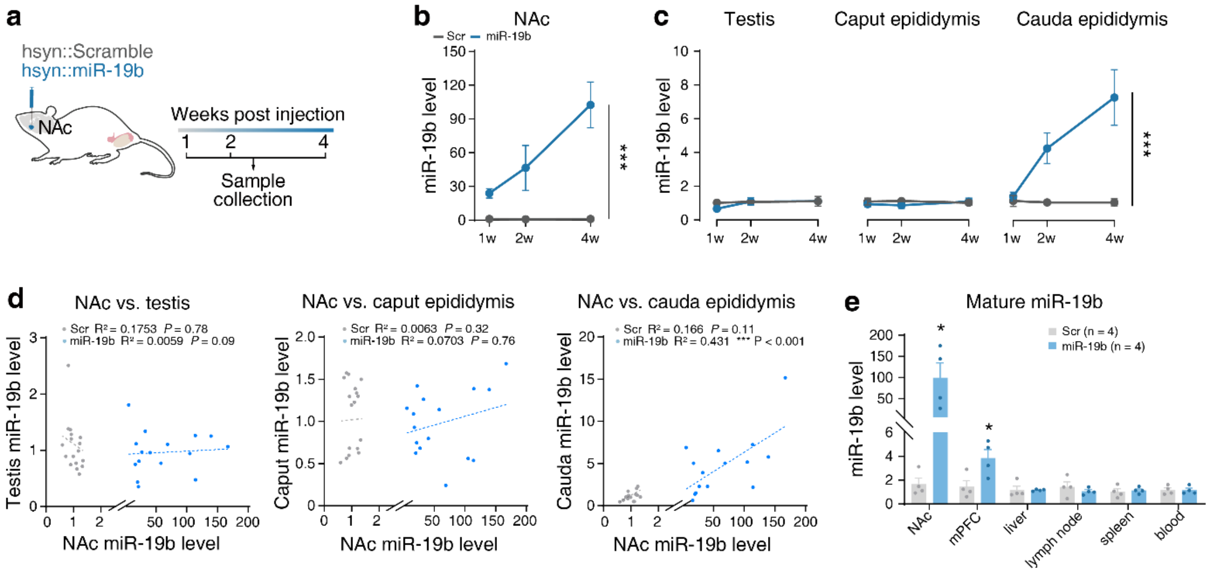

Figure S9. NAc and sperm exhibited correlative miR-19b perturbations. (**a**) Schematic paradigm. AAV-hSyn-Cre and AAV-flex-miR-19b-EGFP or AAV-flex-Scramble-EGFP were injected into the nucleus accumbens of naïve male rats and samples were taken from the indicated tissues after 1, 2, or 4 weeks for qPCR analysis. (**b**) MiR-19b level in NAc. (**c**)Mir-19b level in testis, cauda epididymis and cauda epididymis. n = 3-5 rats for each time point. (**d**) Expression correlation of miR-19b with testis, cauda epididymis and cauda epididymis. (**e**) Mir-19b level in various tissues 4 weeks after viral injection. Values are expressed as mean ± s.e.m.*$P < 0.05$, **$P < 0.01$, ***$P < 0.001$.

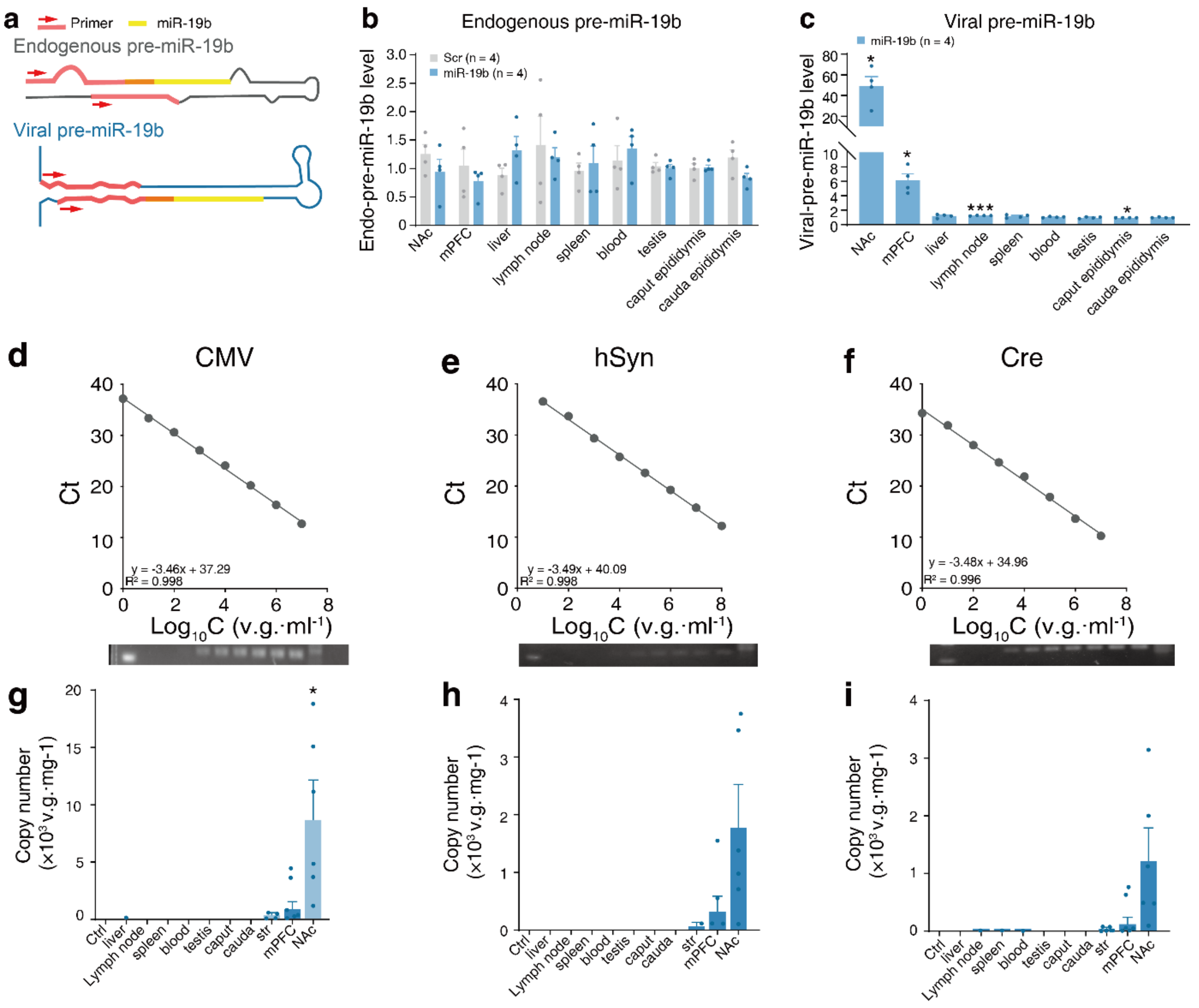

Figure S10. Assessment of potential leakage of AAV-miR-19b. (**a**) The structure of viral and endogenous pre-miR-19b. (**b**) The level of endogenous pre-miR-19b in various tissues 4 weeks after virus injection of AAV-Scr or AAV-miR-19b in nucleus accumbens of naive male rats. (**c**) The level of viral pre-miR-19b in various tissues 4 weeks after virus injection in nucleus accumbens of naive male rats. **(d-f)** Viral DNA was serially diluted in 10-fold gradient to produce the standard curve. (**g-i**) Viral detection in liver, lymph node, spleen, blood, testis, caput, cauda, striatum, mPFC and NAc. The copy number of viruses was calculated as viral particles per mg protein. N = 4 for Scr and miR-19b overexpression each. Values are expressed as mean ± s.e.m.*$P < 0.05$.

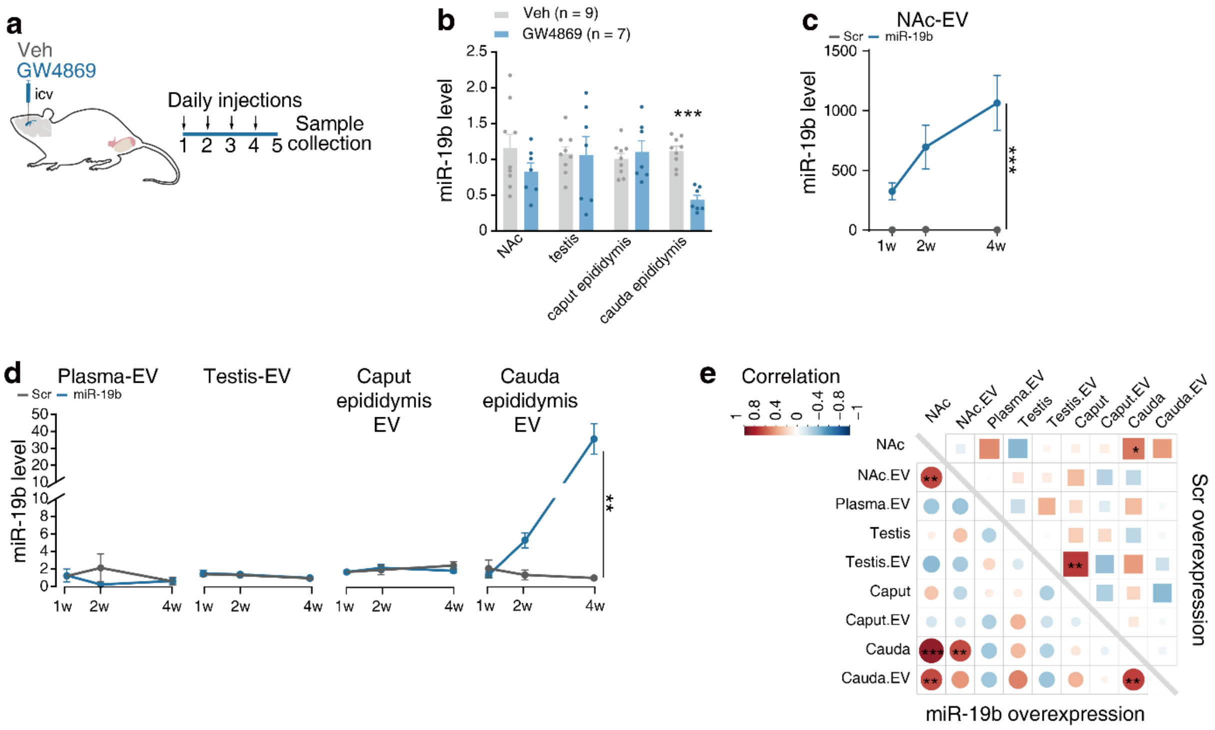

Figure S11. Possible role of extracellular vesicles in miRNA trafficking between NAc and sperm. (**a**) GW4869 or vehicle were injected into the ventricle of naïve male rats and samples were collected after 5 days for qPCR analysis. (**b**) MiR-19b level in NAc, testis, cauda epididymis and cauda epididymis after GW4869 delivery. (**c-d**) Extracellular vesicle miR-19b level in NAc (c), serum, testis, cauda epididymis and cauda epididymis (d) after 1, 2, or 4 weeks. n = 3-5 rats for each time point.(**e**) Correlation plot. The Pearson correlation coefficient of miR-19b expression level between tissue was plotted. Values are expressed as mean ± s.e.m.*P < 0.05, **P < 0.01, ***P < 0.001
